# Regulation of ethylene-related gene expression by indole-3-acetic acid and 4-chloroindole-3-acetic acid in relation to pea fruit and seed development

**DOI:** 10.1093/jxb/erx217

**Published:** 2017-07-11

**Authors:** Charitha P A Jayasinghege, Jocelyn A Ozga, Kosala D Waduthanthri, Dennis M Reinecke

**Affiliations:** Plant BioSystems, Department of Agricultural, Food and Nutritional Science, University of Alberta, Edmonton, Alberta, Canada

**Keywords:** ACC oxidase, ACC synthase, 1-aminocyclopropane-1-carboxylic acid, auxin, 4-chloroindole-3-acetic acid, ethylene biosynthesis and signaling, fruit development, hormonal interaction, indole-3-acetic acid, *Pisum sativum*

## Abstract

In pea, the auxins 4-chloroindole-3-acetic acid (4-Cl-IAA) and indole-3-acetic acid (IAA) occur naturally; however, only 4-Cl-IAA stimulates pericarp growth and gibberellin (GA) biosynthesis, and inhibits the ethylene response in deseeded ovaries (pericarps), mimicking the presence of seeds. Expression of ovary ethylene biosynthesis genes was regulated similarly in most cases by the presence of 4-Cl-IAA or seeds. *PsACS1* [which encodes an enzyme that synthesizes 1-aminocyclopropane-1-carboxylic acid (ACC)] transcript abundance was high in pericarp tissue adjacent to developing seeds following pollination. ACC accumulation in 4-Cl-IAA-treated deseeded pericarps was driven by high *PsASC1* expression (1800-fold). 4-Cl-IAA, but not IAA, also suppressed the pericarp transcript levels of *PsACS4*. 4-Cl-IAA increased *PsACO1* and decreased *PsACO2* and *PsACO3* expression (enzymes that convert ACC to ethylene) but did not change ACO enzyme activity. Increased ethylene was countered by a 4-Cl-IAA-specific decrease in ethylene responsiveness potentially via modulation of pericarp ethylene receptor and signaling gene expression. This pattern did not occur in IAA-treated pericarps. Overall, the effect of 4-Cl-IAA and IAA on ethylene biosynthesis gene expression generally explains the ethylene evolution patterns, and their effects on GA biosynthesis and ethylene signaling gene expression explain the tissue response patterns in young pea ovaries.

## Teaser

Modulation of ethylene biosynthesis and signaling pathways is a key node for seed and auxin regulation of pea fruit growth and development.

## Introduction

The role of ethylene during fruit ripening in climacteric fruit is well known and extensively characterized (Klee and Giovannoni, 2011); however, only limited information is available on ethylene’s role during early fruit development. Pea (*Pisum sativum* L.) is a useful model system to study the role of plant hormones in early fruit set and development (Orzáez *et al.*, 1999; Johnstone *et al.*, 2005; Ozga *et al.*, 2009). Pea flowers undergo self-pollination and ovule fertilization by the time of flower opening (0 days after anthesis; 0 DAA; Cooper, 1938), leading to fruit set and rapid development of the ovary. Ethylene evolution from the pea ovary decreased following pollination and fertilization, while it increased in non-pollinated ovaries that will senesce, implicating ethylene in the senescence of non-pollinated fruits (Orzáez *et al.*, 1999).

During ethylene biosynthesis, *S*-adenosylmethionine (SAM) is converted to 1-aminocyclopropane-1-carboxylic acid (ACC) by ACC synthase (ACS), and ACC is oxidized to ethylene by ACC oxidase (ACO; Bürstenbinder and Sauter, 2012). The step catalyzed by ACS is usually considered the major regulatory step in ethylene biosynthesis; however, the step catalyzed by ACO may regulate the rate of ethylene biosynthesis in certain physiological processes (Dorling and McManus, 2012). In both cases, the transcriptional regulation of *ACS* and *ACO* genes is a major determinant of enzyme levels (Xu and Zhang, 2015). ACS and ACO are encoded by small gene families. In Arabidopsis (*Arabidopsis thaliana*), eight functional *ACS* and five *ACO* genes have been identified (Dorling and McManus, 2012; Harpaz-Saad *et al.*, 2012). Different *ACS* and *ACO* gene family members can become transcriptionally active depending on conditions such as tissue type, developmental stage, or environmental stimuli (Dorling and McManus, 2012; Harpaz-Saad *et al.*, 2012). Auxin is a well-known inducer of ethylene biosynthesis by increasing the transcript abundance of *ACS* (Peck and Kende, 1998; Harpaz-Saad *et al.*, 2012) and, in some instances, *ACO* (Chae *et al.*, 2000) genes. In Arabidopsis, all of the functional *ACS* genes have been reported to be induced by indole-3-acetic acid (IAA) to some extent in seedling or root tissues (Tian *et al.*, 2002; Yamagami *et al.*, 2003; Tsuchisaka and Theologis, 2004; Lee *et al.*, 2009). The presence of at least one auxin response element (AuxRE) within the 2 kb promoter region upstream of the 5′ start codon has been reported for *AtACS4*, *AtACS6*, *AtACS8*, *AtACS9*, and *AtACS11* (Lee *et al.*, 2009), and data also support the presence of AuxREs in the 5′-promoter regions of *ACO* genes (Khodakovskaya *et al.*, 2006; Yuan and Dean, 2010). Apart from the regulation of ethylene production at the ACC synthesis and oxidation steps, ACC can undergo conjugation mainly to malonyl-ACC (MACC; Dorling and McManus, 2012), limiting its availability for ethylene biosynthesis.

Ethylene must be perceived and its signal transduced for plant tissues to elicit an ethylene response. In Arabidopsis, there are five ethylene receptors ETHYLENE RESPONSE 1 (ETR1), ETR2, ETHYLENE RESPONSE SENSOR 1 (ERS1), ERS2, and ETHYLENE INSENSITIVE 4 (EIN4; Hua *et al.*, 1998). In the absence of ethylene, ethylene receptors repress ethylene signaling through the activation of the negative regulator CONSTITUTIVE TRIPLE-RESPONSE 1 (CTR1; Hua and Meyerowitz, 1998). CTR1 is a Raf-like protein kinase that phosphorylates the downstream signaling component EIN2, leading to the destabilization of EIN3/ETHYLENE INSENSITIVE 3-LIKE 1 (EIL1) transcription factors, preventing the expression of ethylene-responsive genes (Qiao *et al.*, 2012). Ethylene-bound receptors no longer activate CTR1, which prevents phosphorylation of EIN2 by CTR1. This causes the cleavage of the C-terminal domain of EIN2 (EIN2C), which is subsequently translocated to the nucleus, where it stabilizes EIN3, allowing the expression of ethylene-responsive genes (Qiao *et al.*, 2012). The levels of EIN3/EIL1 are regulated by two F-box proteins EIN3 BINDING F-BOX1 (EBF1) and EBF2, which mediate EIN3/EIL1 degradation through a proteasome-mediated pathway (Gagne *et al.*, 2004). EIN2C is also involved in the inhibition of EBF1/2 translation in the cytosol (Li *et al.*, 2015; Merchante *et al.*, 2015).

Transcriptomic studies in tomato indicate that a number of putative ethylene, auxin, gibberellin (GA), and abscisic acid biosynthesis and signaling-related genes are differentially expressed in response to pollination and during early fruit development (Vriezen *et al.*, 2008; Wang *et al.*, 2009). However, the mechanisms of interaction between these classes of hormone signaling molecules to bring about fruit growth and development were not explored in these global transcriptome studies. In pea, GAs and/or auxins such as 4-chloroindole-3-acetic acid (4-Cl-IAA) have been implicated as hormonal signals co-ordinating pericarp growth (Eeuwens and Schwabe, 1975; Ozga *et al.*, 1992, 2003, 2009). 4-Cl-IAA and IAA are naturally occurring auxins found in developing pea seeds (Marumo *et al.*, 1968; Magnus *et al.*, 1997; Reinecke, 1999). 4-Cl-IAA treatment to deseeded pea pericarps can mimic the presence of seeds with respect to growth, suggesting that this auxin may act as a seed-derived signal promoting fruit growth (Ozga and Reinecke, 1999; Ozga *et al.*, 2002, 2009). However, the ubiquitously occurring auxin, IAA, was incapable of rescuing deseeded pericarp growth (Reinecke *et al.*, 1995). Furthermore, seeds and 4-Cl-IAA, but not IAA, were capable of regulating the transcript abundance of specific pericarp GA biosynthesis and metabolism genes that increase or maintain bioactive GA_1_ levels in the pericarp, which can induce pericarp growth (Ozga *et al.*, 2009). These data support the working hypothesis that seed-derived auxin (4-Cl-IAA in pea) is transported from seeds to the pericarp, where it stimulates GA biosynthesis and pericarp growth (Ozga *et al.*, 2009).

The ability of 4-Cl-IAA, but not IAA, to stimulate pericarp GA biosynthesis is probably a major factor in 4-Cl-IAA-mediated pericarp growth. As well, 4-Cl-IAA minimizes the growth-inhibitory effects of ethylene on pericarp tissue, while IAA does not (Johnstone *et al.*, 2005). Reinecke *et al.* (1995) found that although 4-Cl-IAA stimulated deseeded pericarp growth at 1–100 µM, IAA at ≥10 µM inhibited deseeded pericarp growth. As a pre-treatment with the ethylene action inhibitor silver thiosulfate (STS) blocked the IAA-induced inhibition of deseeded pericarp growth, IAA-induced ethylene was implicated as the growth-inhibitory factor in the pericarp assays (Johnstone *et al.*, 2005). The difference in growth response between 4-Cl-IAA and IAA was not attributed to differences in auxin-induced ethylene evolution, as both auxins stimulated similar ethylene evolution profiles when applied to deseeded pea pericarps (Johnstone *et al.*, 2005). Furthermore, application of the ethylene-releasing agent ethephon to 4-Cl-IAA-treated deseeded pericarps had minimal effect on pericarp growth (Johnstone *et al.*, 2005). Overall, these data show that the interaction of these two naturally occurring auxins with ethylene in pea fruit tissue is fundamentally different; however, the specific mechanisms that bring about the differential auxin–ethylene interactions are not known.

In this study, we investigated the hormonal signals, auxin and ethylene, during early fruit growth to determine the type and level of their interaction that influences fruit set and development in pea. Specifically, we investigated the underlying mechanisms involved in seed-, 4-Cl-IAA-, and IAA-specific regulation of the pericarp ethylene biosynthesis and signaling pathways at the transcriptional level, and used the ethylene action inhibitor STS and the ethylene-releasing agent ethephon as tools to test their transcriptional regulation. These transcriptional profiles were correlated with ACC production, ACC oxidase activity, previously reported ethylene evolution levels, and pericarp growth. From these studies, we developed a model of auxin and ethylene regulation of early pea fruit development that supports the working hypothesis that auxin (4-Cl-IAA in pea) produced in the developing seeds stimulates growth and development in the surrounding ovary by regulating a network of hormonal pathways in the ovary including the modulation of ethylene biosynthesis and response.

## Materials and methods

### Plant material and experimental procedures

The *Pisum sativum* L. cv. I_3_ (Alaska-type) cultivar was used in all experiments. Mature dry pea seeds were planted in 3 liter pots containing a 4:1 (v/v) mixture of Sunshine Mix #4/LA4 (SunGro Horticulture, MA, USA) and sand. Plants (three per pot) were grown in a growth chamber under cool white fluorescent lights with an average photon flux density of 240 µE m^–2^ s^–1^ and 16 h photoperiod (light, 19 °C; dark, 17 °C). The main shoot apex remained intact, while expanding lateral shoots were removed as they developed.

For the developmental study, fruits from flowering nodes 1–6 were tagged at the date of anthesis (0 DAA) and pericarps within a specific length range were used (1 DAA, 8–12 mm; 2 DAA, 15–20 mm; 3 DAA, 26–33 mm). For non-pollinated fruits, floral buds at –2 DAA were emasculated and tissues were collected at –2, 0, 1, 2 and 3 DAA. Fruits were collected onto ice and immediately dissected into seed/ovule, pericarp wall, pericarp dorsal vascular suture, and pericarp ventral vascular suture tissues (see pericarp tissue, Supplementary Fig. S1C at *JXB* online), except for those at –2 DAA, where the ovules were removed from the fruit, and the pericarps with the stigma and style attached were harvested. Tissues were frozen in liquid N_2_ immediately after harvest and dissection, and stored at –80 °C.

For the hormonal application studies, one fruit per plant from the third to sixth flowering nodes was used; the remaining flowers were removed. The pericarps remained attached to the plant during the entire experiment. Hormonal treatments were applied to the pericarps using the split-pod technique (Ozga *et al.*, 1992). Fruits at 2 DAA measuring 15–20 mm in length were split down the dorsal suture and seeds were either left intact (SP) or removed (SPNS). Surgical manipulation of the fruit was completed 12 h prior to solution application, with one exception. STS was applied to the pericarp immediately after splitting and deseeding, with subsequent hormonal application occurring 12 h after STS application. All solutions (30 μl) were applied directly to the inside surface of the pericarp wall (endocarp). Deseeded pericarps were treated with IAA or 4-Cl-IAA (50 μM in 0.1% aqueous Tween-80), ethephon (1000 mg l^–1^ in 0.1% aqueous Tween-80), or STS (1 mM in 0.1% aqueous Tween-80) alone or in combination. Auxin or auxin–ethephon combinations were IAA plus 4-Cl-IAA, IAA plus ethephon, or 4-Cl-IAA plus ethephon, in 0.1% aqueous Tween-80. Auxin or ethephon combinations with STS were IAA plus STS, 4-Cl-IAA plus STS, or ethephon plus STS, with a final concentration of auxins at 50 μM, ethephon at 1000 mg l^–1^, and STS at 1 mM in 0.1% aqueous Tween-80. The SP and SPNS controls were treated with 0.1% aqueous Tween-80. When fruits were treated with both auxin and ethephon, ethephon was applied 90 min after the auxin treatment and samples were collected based on the time after auxin treatment. All surgically modified pericarps were covered with plastic bags to maintain high humidity throughout the experiment. Pericarps were harvested at 0, 2, 8, and 12 h after solution application (or 12, 14, 20, and 24 h after pericarp splitting or splitting and deseeding). Fruits were harvested and immediately transferred into liquid N_2_, and subsequently stored at –80 °C.

### RNA isolation

Frozen tissues were ground in liquid N_2_ to a fine powder, and ~40–60 mg of ground tissue per sample was used for total RNA isolation using a modified TRIzol (Life Technologies) method as described in Ayele *et al.* (2006). Total RNA concentration was measured at *A*_260_ using a NanoDrop (ND-1000 or 2000c) spectrophotometer, and RNA quality and integrity were estimated using OD_260_/OD_280_ and OD_260_/OD_230_ ratios and gel electrophoresis, or an Agilent 2100 bioanalyzer. Given high RNA quality, the total RNA was DNase treated (Ambion DNA-free kit) as per the manufacturer’s protocol. Following DNase treatment, the concentration of the total RNA samples was requantified at *A*_260_ using a NanoDrop (ND-1000 or 2000c) spectrophotometer and then diluted with nuclease-free water to obtain a concentration of 40 ng µl^–1^. The accuracy of the dilution was reverified at *A*_260_, and then the total RNA samples were stored at –80 °C.

### qPCR assays

For profiling ethylene biosynthesis gene expression in pea, *PsACS1*, *PsACS2*, and *PsACO1* mRNA coding sequences available in GenBank (accession nos AF016458, AF016459, and M98357, respectively); and *PsACS4*, *PsACO2*, and *PsACO3* mRNA coding sequences isolated and sequenced from *P. sativum* L. cv. I_3_ for this study (GenBank accession nos KX255646, KX261617, and KX261618, respectively, see Supplementary Protocol S1) were used. Marker genes profiled for the ethylene signaling pathway (selected based on previous observations that they become transcriptionally active in response to ethylene; Hua *et al.*, 1998; Kevany *et al.*, 2007; Konishi and Yanagisawa, 2008; Yang *et al.*, 2010) were two putative ethylene receptor genes, *PsERS1* (GenBank: AF039746) and *PsETR2* (GenBank: KX261619), and two putative genes that code for F-box proteins that are negative regulators of ethylene signal transduction, *PsEBF1* (GenBank: KX261620) and *PsEBF2* (GenBank: KX261621). *PsETR2*, *PsEBF1*, and *PsEBF2* were isolated and sequenced from *P. sativum* L. cv. I_3_ for this study (see Supplementary Protocol S1). Primers and probes for qPCR analysis for *PsACS1*, *PsACS2*, *PsACS4*, *PsACO1*, *PsACO2*, *PsACO3*, *PsERS1*, *PsETR2*, *PsEBF1*, and *PsEBF2* were designed with Primer Express Software (Version 3.0; Life Technologies) using the non-minor groove binding option. The ethylene biosynthesis and signaling gene probes were double-quenched (Integrated DNA Technologies) with an Iowa Black FQ (IBFQ) quencher at the 3′ end, and a ZEN quencher positioned 9 bp from the 6-FAM fluorescent dye-containing 5′ end (Supplementary Table S1). Primers and probe for the 18S rRNA control were designed by Ozga *et al.* (2003). For all transcript targets, reverse transcription and quantification were performed as one-step reactions with a TaqMan One-Step RT-PCR Master Mix Reagents Kit or a TaqMan RNA-to-Ct 1-Step Kit (Applied Biosystems) in a StepOnePlus Real-Time PCR system (Applied Biosystems) as described in Ayele *et al.* (2006) and Reinecke *et al.* (2013; see Supplementary Protocol S2).

### ACC content and ACO enzyme activity analyses

Pericarp samples of 4-Cl-IAA- and IAA-treated fruits were collected 12 h after hormone treatment, and the controls (intact, SP, and SPNS) were collected at the equivalent time point (24 h after pericarp splitting and seed removal). Pericarps were immediately frozen in liquid N_2_, and then stored at –80 °C. The ACC and ACC conjugate content, and ACO enzyme activity were determined as described by Bulens *et al.* (2011) with modifications. For ACC analysis, 4.5 ml of 5% (w/v) aqueous sulfosalicylic acid was added to ground pericarp tissue (1 g), vortexed, then gently shaken at 4 °C for 30 min, followed by centrifugation at 4284 *g* for 10 min at 4 °C. The supernatant (ACC extract) was frozen in liquid N_2_ and stored at –80 °C. For free ACC analysis, the ACC extract (1.4 ml) was thawed on ice and mixed with 400 µl of 10 mM aqueous HgCl_2_ in a 7 ml glass vial closed with a rubber septum. Through the septum, 200 µl of NaOCl–NaOH mixture (2:1, v/v) was injected and vortexed for 5 s. After 4 min of incubation on ice and vortexing for 5 s, a headspace sample was taken for gas chromatograph (GC) analysis (see Supplementary Protocol S3).

For total ACC analysis, the ACC extract (500 µl) was thawed on ice, mixed with 200 µl of 6 M HCl, and incubated in a boiling water bath for 3 h before neutralizing with 200 µl of 6 M NaOH. The supernatant containing total ACC was separated by centrifugation (21 000 *g* for 6 min), and 100 µl of the supernatant was diluted with 600 µl of deionized water. The diluted extract was used for total ACC analysis as described for the free ACC analysis, but with half-size reaction volumes. ACC conjugate was estimated by subtracting free ACC from the total ACC content within a sample.

For ACO enzyme activity analysis, ground plant tissue (500 mg) was mixed with 50 mg of polyvinylpolypyrrolidone and 1 ml of MOPS extraction buffer [400 mM MOPS, 30 mM ascorbic acid sodium salt, and 10% (v/v) glycerol; pH 7.2], and incubated in a 4 °C water bath for 10 min with gentle shaking. The supernatant was isolated by centrifugation (21 000 *g* for 30 min at 4 °C) and frozen in liquid N_2_, then stored at –80 °C. For analysis, the extract was thawed on ice and 400 µl was placed into a 7 ml glass vial with 3.6 ml of MOPS reaction buffer [50 mM MOPS, 5 mM ascorbic acid sodium salt, 10% (v/v) glycerol, 20 mM sodium bicarbonate, 0.02 mM iron sulfate, 1 mM ACC, 1 mM DTT; pH 7.2]. The vial was immediately closed with a rubber septum, vortexed for 5 s, incubated in a 30 °C water bath for 1 h while gently shaking, vortexed again for 5 s, and a headspace sample was immediately taken to quantify ethylene produced by the ACO enzyme using GC analysis. At the beginning and end of each set, a blank reaction was done, replacing the supernatant extract with sterile deionized water.

### Statistical analyses

The data means in all experiments are the average of biological replicates (the number of biological replicates is given for each experiment within the specific data set). Probability values (*P*-values) were calculated using a one-tailed *t*-test (assuming unequal variance). Statistical significance was declared at *P*≤0.05 for comparisons between the means.

## Results and Discussion

### Working model

**Fig. 1.**
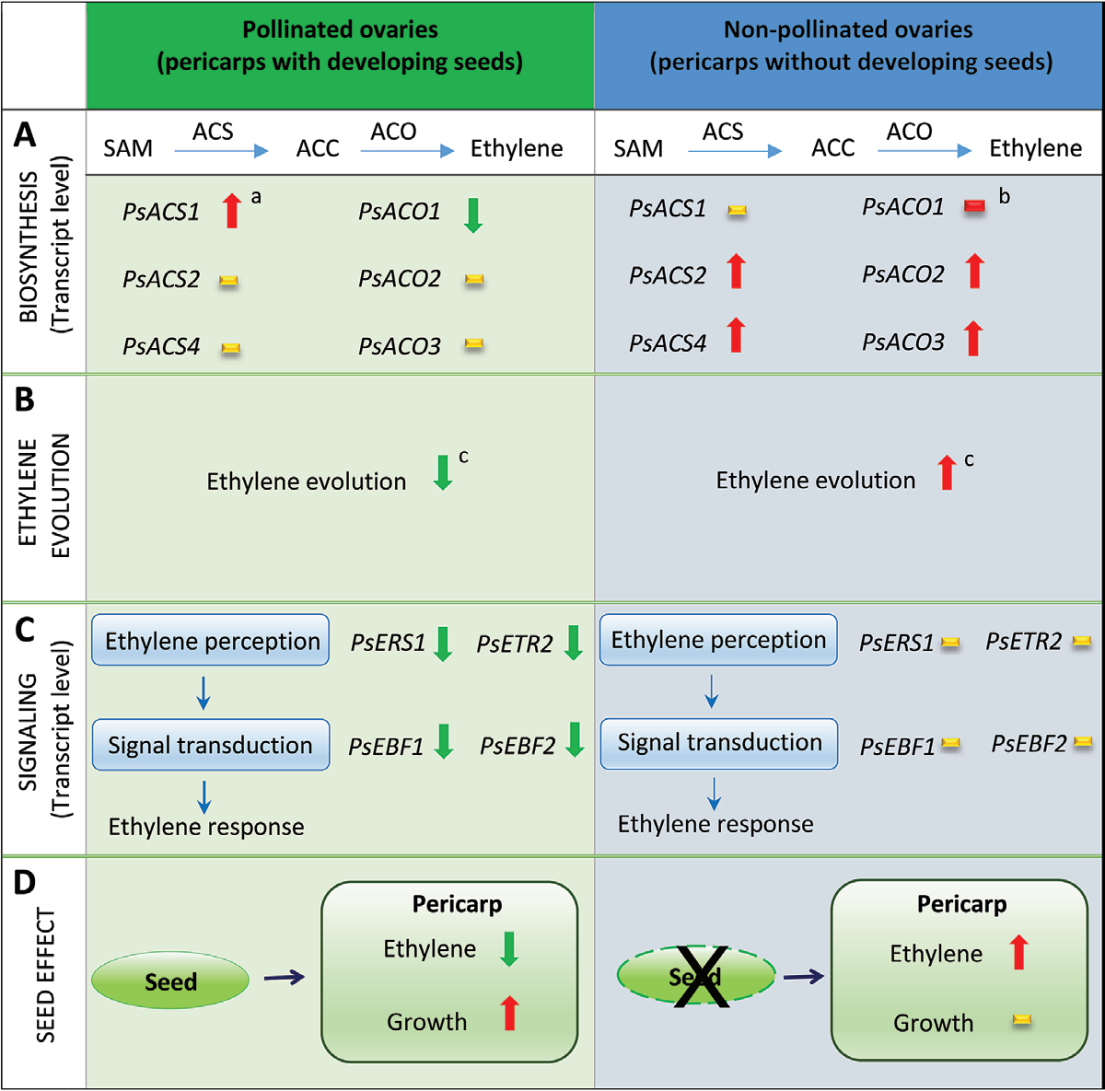
Working model of the effect of developing seeds on ethylene biosynthesis (A and B) and response (C) in pea pericarp tissue from pollinated and non-pollinated ovaries (D). Arrows show an increase (red) and decrease (green) of a given target or response. Minimal to no response is represented by a yellow dash. ^a^Primarily in ventral vascular suture; ^b^transcript abundance remained relatively stable in the ventral vascular suture (red dash); ^c^as reported by Orzáez *et al.* (1999).

**Fig. 2.**
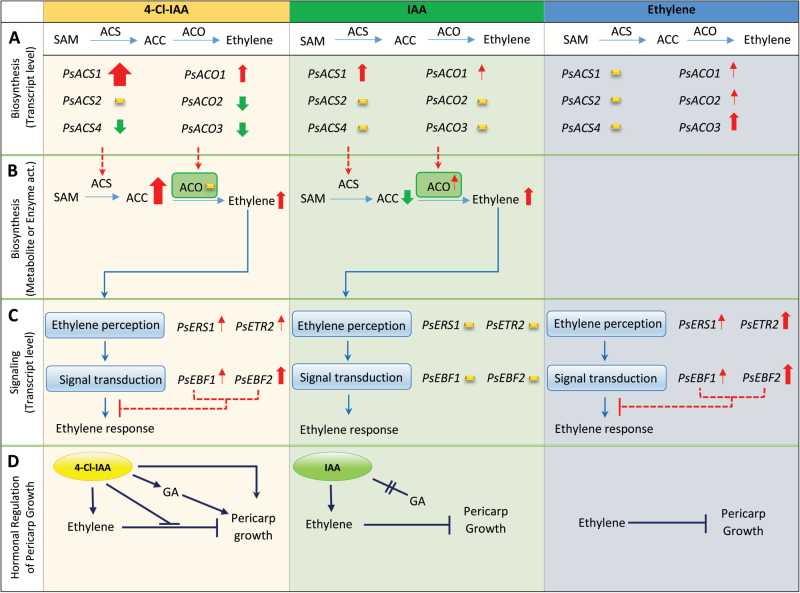
Working model of auxin–ethylene interaction during early pea fruit development. 4-Cl-IAA stimulated deseeded pericarp growth and inhibited the increase in transcript abundance of the pericarp ethylene biosynthesis genes *PsACS4*, *PsACO2*, and *PsACO3*, mimicking the presence of seeds, but IAA did not (A). ACC accumulation in 4-Cl-IAA-treated pericarps was driven by high *PsASC1* expression and changes in *PsACO* expression that did not increase ACO enzyme activity; this pattern did not occur in IAA-treated pericarps (B). Both ethylene and 4-Cl-IAA diminish ethylene signaling output by up-regulating the expression of the pericarp ethylene receptor and signaling-related (EBF) genes (C). Overall, these data support the working hypothesis that auxin (4-Cl-IAA in pea) produced in the developing seeds stimulates growth and development in the surrounding ovary by regulating a network of hormonal pathways in the ovary including stimulation of gibberellin biosynthesis (Ozga *et al.*, 2009) and modulation of ethylene biosynthesis and response (D). Arrows show an increase (red) and decrease (green) of a given target or response, with arrow thickness representing response magnitude. Minimal to no response is represented by a yellow dash.

Here we propose a working model of hormonal interaction during early pea fruit development with specific focus on auxin–ethylene interactions. Following pollination and fertilization events, developing seeds stimulate ovary (pericarp) growth. In this model, auxin (4-Cl-IAA) is proposed as a signaling molecule originating from the seeds that is required for continued fruit development by modulating auxin-specific pathways, as well as GA and ethylene hormonal pathways in the surrounding tissue (Johnstone *et al.*, 2005; Ozga *et al.*, 2009). The following experiments were designed to explore further the interaction of auxin with the ethylene biosynthesis and signaling pathways in developing pea fruit. A model from the data to be described below is presented in Figs 1 and 2 to facilitate interpretations of the results as they are presented.

### Ethylene biosynthesis genes in pea

Phylogenetic analysis of the predicted PsACS1, PsACS2, and PsACS4 proteins demonstrates that these pea ACSs have key sequence homology with functional ACS enzymes in Arabidopsis (Yamagami *et al.*, 2003; Supplementary Protocol S4; Supplementary Figs S2, S3). ACS proteins are divided into three types based on their C-terminal sequences, and ACS sequence analysis categorizes PsACS2 and PsACS4 as type I and PsACS1 as type II ACSs (Xu and Zhang, 2015; see Supplementary information; Supplementary Figs S2, S3).

The ACO proteins that catalyze the oxidation of ACC to ethylene are encoded by a small family of genes (usually 3–4) that belong to a large superfamily of ferrous-dependent non-heme oxygenases (Ruduś *et al.*, 2013). Phylogenetic analysis clusters PsACO1, PsACO2, and PsACO3 with functional ACC oxidases, and all three proteins contain amino acids known to be important for ACO activity (Dilley *et al.*, 2013; Booker and DeLong, 2015; see Supplementary information; Supplementary Protocol S4; Supplementary Figs S4, S5).

### Transcriptional control of pericarp PsACS and PsACO genes by pollination events

**Fig. 3.**
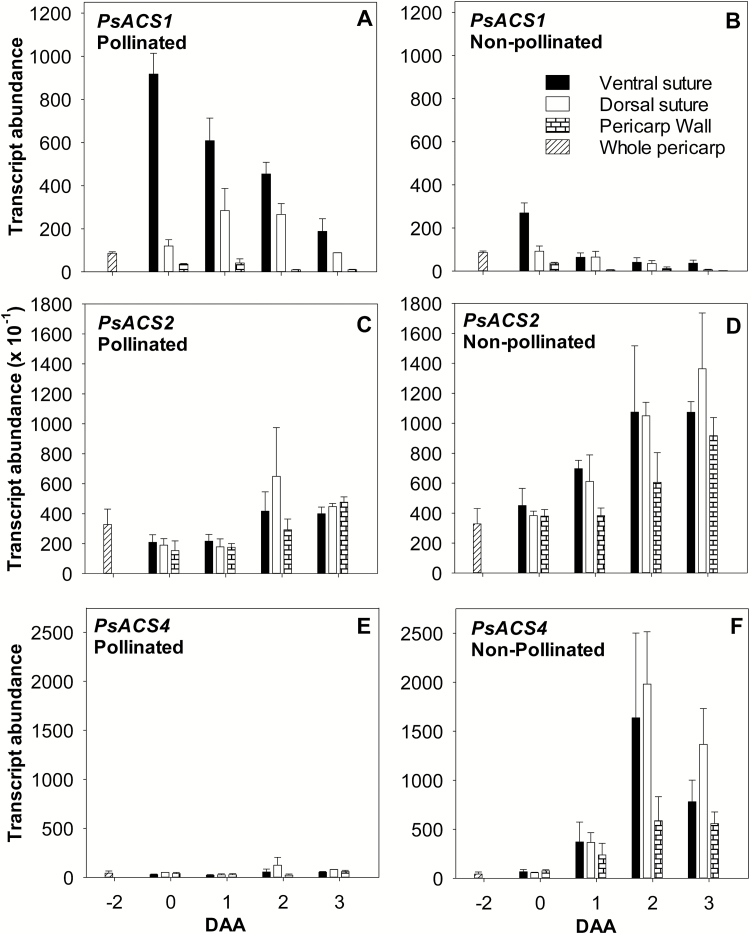
Relative transcript abundance of the ethylene biosynthesis genes *PsACS1* (A and B), *PsACS2* (C and D), and *PsACS4* (E and F) in pollinated and non-pollinated pea fruits. Whole pericarps were assessed at –2 DAA, and pericarp tissues (ventral vascular suture, dorsal vascular suture, and wall) were assessed at 0–3 DAA. For non-pollinated fruits, flowers were emasculated at –2 DAA to prevent pollination. Data are means ±SE, *n*=3, with *n*=2 for a few samples where the tissues were limited due to small tissue size. Each sample is composed of a minimum of four pericarp tissues.

Following pollination and fertilization (0 DAA), the pericarp of pea fruit develops rapidly in length and fresh weight. Minimal growth occurred in the pericarps of non-pollinated ovaries (Supplementary Fig. S1A, B); however, the non-pollinated pericarps were still turgid and green at 3 DAA. High *PsACS1* transcript levels occurred in the pericarp ventral vascular suture of pollinated ovaries at 0 DAA where the developing seeds were attached, and they gradually decreased with fruit development (Fig. 3A; Supplementary Fig. S1C). In the absence of pollination, the change in pericarp *PsACS1* transcripts in the ventral vascular suture was relatively minor, and pericarp *PsACS1* transcript levels were generally low compared with pollinated pericarps (Fig. 3B). As *PsACS1* expression is auxin (IAA) responsive (Peck and Kende, 1995; see below) and young pea seeds are enriched in auxins (IAA and 4-Cl-IAA; Magnus *et al.*, 1997), the pollination-specific pattern of pericarp *PsACS1* expression may be dictated by auxin gradients in the fruit. In contrast to *PsACS1*, transcript abundance of *PsACS2* (Fig. 3C, D) and *PsACS4* (Fig. 3E, F) increased in non-pollinated pericarp tissues compared with pollinated fruits (~2- to 3-fold and 10- to 15-fold, respectively, in pericarp wall and ventral suture by 3 DAA). *PsACS2* transcript levels in the ovules of non-pollinated fruits were also ~2-fold higher compared with seeds of pollinated fruits (Supplementary Table S2). These data suggest that *PsACS1* expression could be associated with auxin-related fruit set events, and *PsACS2* and *PsACS4* are up-regulated as part of an ethylene-related senescence process (Fig. 1A).

**Fig. 4.**
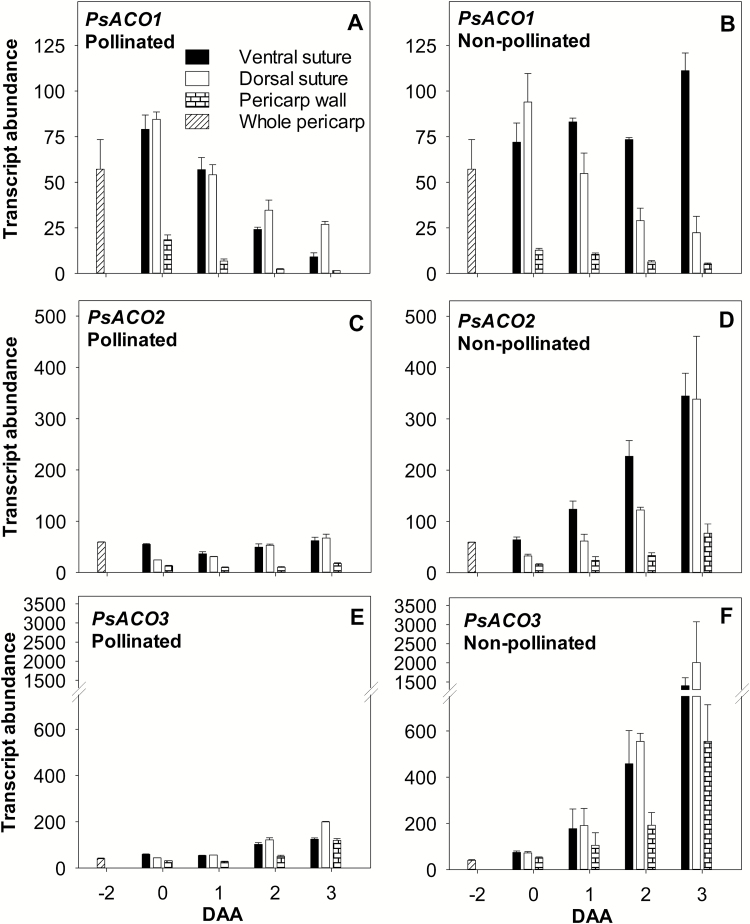
Relative transcript abundance of the ethylene biosynthesis genes *PsACO1* (A and B), *PsACO2* (C and D), and *PsACO3* (E and F) in pollinated and non-pollinated pea fruits. Whole pericarps were assessed at –2 DAA, and pericarp tissues (ventral vascular suture, dorsal vascular suture, and wall) were assessed at 0–3 DAA. For non-pollinated fruits, flowers were emasculated at –2 DAA to prevent pollination. Data are means ±SE, *n*=3, with *n*=2 for a few samples where the tissues were limited due to small tissue size. Each sample is composed of a minimum of four pericarp tissues.

The transcripts of *PsACO1*, *PsACO2*, and *PsACO3* were more abundant in the pericarp vascular sutures than in the wall (Fig. 4; Supplementary Table S2). In the absence of pollination, *PsACO1* transcript abundance remained elevated in the pericarp ventral suture of non-pollinated ovaries, while the transcript level decreased in the other tissues as observed in pollinated pericarps (Fig. 4A, B). In contrast to *PsACO1*, *PsACO2* and *PsACO3* transcript abundance increased (2- to 5-fold and 5- to 11-fold, respectively) in all pericarp tissues of non-pollinated ovaries compared with those of pollinated ovaries by 2–3 DAA (compare Fig. 4C with 4D and 4E with 4F) suggesting that up-regulation of these genes is involved in programed senescence of the ovary.

Overall, elevated transcript abundance of *PsACS2*, *PsACS4*, *PsACO2*, and *PsACO3* in the pericarps of non-pollinated ovaries (Fig. 1A) implies that, in the absence of pollination, the co-ordinated up-regulation of expression of these genes increases the capacity to produce ACC and convert ACC to ethylene, facilitating ovary senescence. This is consistent with elevated ethylene evolution from non-pollinated pea ovaries by 2 DAA compared with pollinated ovaries (Orzáez *et al.*, 1999). The expression patterns of *PsACS1* and *PsACO1* genes indicate that these genes are not directly associated with the increased ethylene evolution and senescence in non-pollinated fruits.

### Effect of seeds on pericarp ethylene biosynthesis during fruit set

**Fig. 5.**
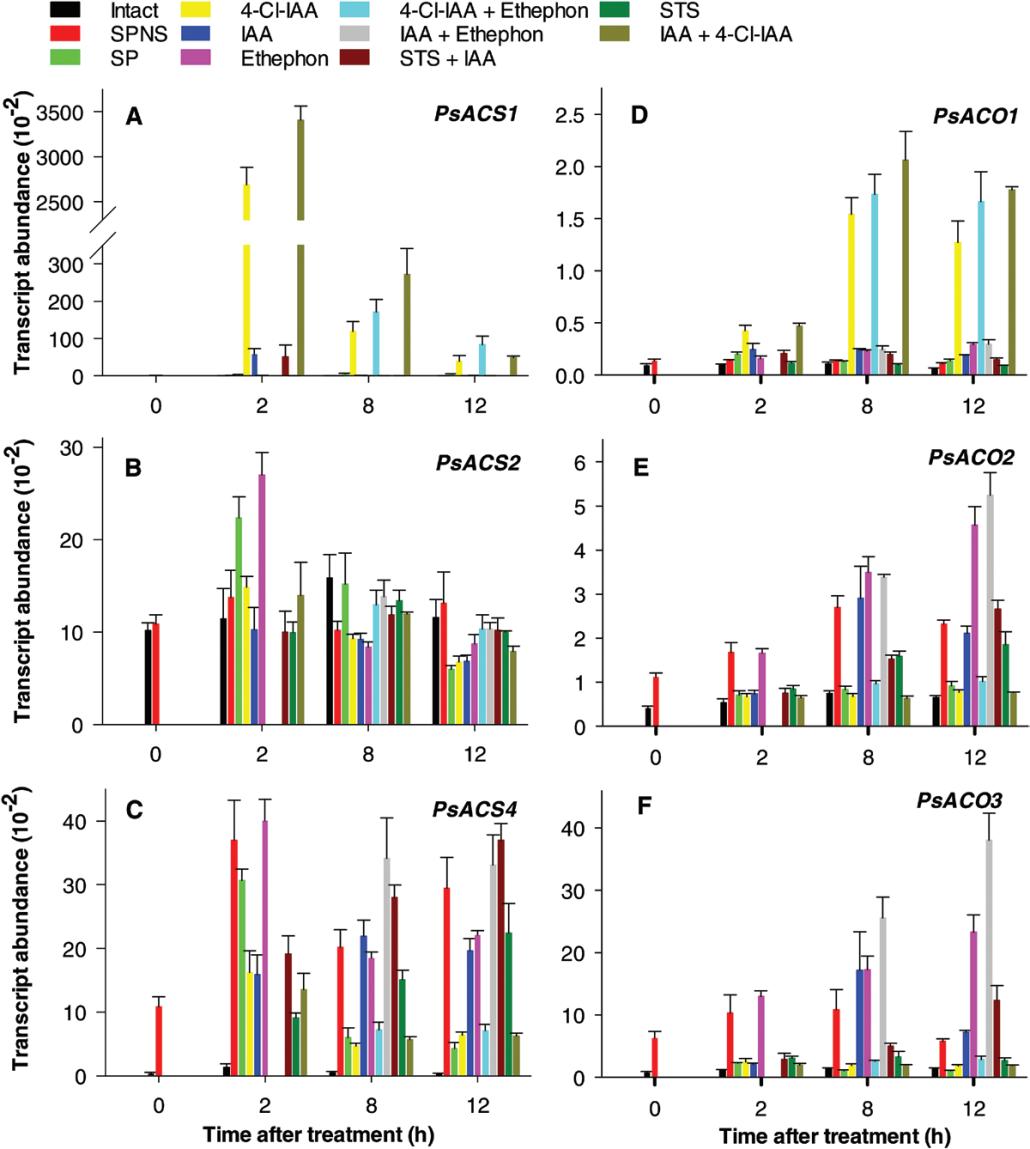
Effect of seed removal and hormone treatment on the relative transcript abundance of the ethylene biosynthesis genes *PsACS1* (A), *PsACS2* (B), *PsACS4* (C), *PsACO1* (D), *PsACO2* (E), and *PsACO3* (F) in the pericarps of pollinated pea ovaries. Pericarps at 2 DAA were either left intact, split (SP), or split and deseeded (SPNS), and treated 12 h after deseeding with 4-Cl-IAA, IAA, or ethephon alone or in combination. When fruits were treated with both auxin and ethephon, ethephon was applied 90 min after the auxin treatment and samples were collected based on the time after auxin treatment. Because of the delayed ethephon application, the auxin- plus ethephon-treated pericarps were not studied at the 2 h time point. Deseeded pericarps were also pre-treated with STS at pericarp splitting and deseeding (STS treatment), and IAA was applied to STS-pre-treated pericarps (IAA plus STS treatment). The SP and SPNS controls were treated with 0.1% aqueous Tween-80. All the samples were collected with respect to the time after hormone treatment. Data are means ±SE, *n*=3–8, with the exception of STS+IAA 2 h treatment, where *n*=2.

**Fig. 6.**
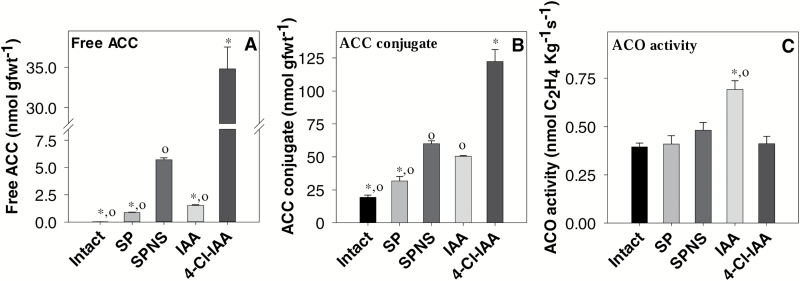
Free ACC (A), ACC conjugate (B), and ACO enzyme activity (C) of pea pericarps with seeds and deseeded pericarp treated with IAA or 4-Cl-IAA. Pea pericarps were intact, split (SP), or split and deseeded (SPNS), and treated with 50 μM IAA or 4-Cl-IAA in aqueous 0.1% Tween-80, or aqueous 0.1% Tween-80 (SPNS control) 12 h after deseeding. Assays were completed on tissues 12 h after the hormone treatment. Data are means ±SE *n*=3, except for intact, SP, and SPNS free ACC and ACC conjugate analysis, where *n*=2. Comparisons were made using student’s *t*-test. *Means are significantly different from that of SPNS at *P*<0.05. °Means are significantly different from that of 4-Cl-IAA at *P*<0.05.

Pea pericarps with seeds (intact or SP) will continue to grow; however, seed removal will inhibit pericarp growth and lead to abscission (Ozga *et al.*, 1992; Supplementary Fig. S6A). Minimal to no changes in the transcript abundance of pericarp *PsACS* (*PsACS1*, *PsACS2*, and *PsACS4*) and *PsACO* (*PsACO1*, *PsACO2*, and *PsACO3*) genes from 2 DAA intact fruits were observed throughout the 12 h experimental period (Fig. 5; Supplementary Fig. S7). Associated with this transcript abundance pattern, intact pea pericarps contained trace amounts of free ACC (~0.03 nmol g FW^–1^; Fig. 6A), a moderate level of ACC conjugate (19 nmol g FW^–1^; Fig. 6B), and evolved low levels of ethylene (7–27 nl g FW^–1^, at 2–3 DAA; Johnstone *et al.*, 2005).

For the split-pericarp assays (Ozga *et al.*, 1992), pericarps were assessed or treated 12 h after splitting to avoid wound-induced ethylene evolution (which peaks ~4 h after pericarp splitting; Johnstone *et al.*, 2005) and allow time for seed-derived factors to diminish in the pericarp tissue in the treatments where the seeds are removed. By 24 h after pericarp splitting (12 h after treatment), the transcript abundances of the *PsACS* and *PsACO* ethylene biosynthesis genes in the pericarps with seeds (SP) were in the general range of that in the intact pericarps, except for that of *PsACS1*, which had increased by 28-fold (Fig. 5; Supplementary Fig. S7). The *PsACS* and *PsACO* transcript abundance patterns in SP pericarps were associated with increased free ACC (0.9 nmol g FW^–1^; 25-fold; Fig. 6A), and ethylene evolution levels (Johnstone *et al.*, 2005) 24 h after pericarp splitting compared with pericarps of intact fruit.

Seed removal (compare SPNS with SP) increased pericarp *PsACS4*, *PsACO2*, and *PsACO3* transcript abundance (7-, 2.5-, and 5-fold, respectively, by 24 h after deseeding) and suppressed the increase in pericarp *PsACS1* transcript abundance (Fig. 5C, E, F; Supplementary Fig. S7A, C, E, F), similar to the *PsACS* and *PsACO* expression patterns observed in the pericarps of non-pollinated ovaries (Fig. 1A). These *PsACS* and *PsACO* gene expression patterns were associated with a 6-fold higher free ACC (5.7 nmol g FW^–1^), 2-fold higher ACC conjugate (60 nmol g FW^–1^; compare SPNS and SP; Fig. 6A, B), and higher ethylene evolution levels (Johnstone *et al.*, 2005) in deseeded pea pericarps compared with pericarps with seeds. As the ACO enzyme activity was similar in pericarps with (SP) and without seeds (SPNS; 24 h after pericarp splitting; Fig. 6C), it is likely that the increase in ACC levels was the primary factor in increasing ethylene evolution in deseeded pericarps which will subsequently senesce. For comparison, pericarp ACC levels in the intact pollinated pea fruit are similar to those of pre-climacteric fruit of banana (<0.05 nmol g FW^–1^), and ACC levels in the deseeded pea pericarp (SPNS) are similar to those of ripening banana fruit (5 nmol g FW^–1^; Hoffman and Yang, 1980).

### 4-Cl-IAA and IAA differentially regulate pericarp ethylene biosynthesis

4-Cl-IAA stimulated deseeded pea pericarp growth and IAA did not (Reinecke *et al.*, 1995; Supplementary Fig. S6). We originally expected that both auxins (4-Cl-IAA and IAA) would affect ethylene biosynthesis gene expression in a similar fashion, as the ethylene evolution profiles were similar from 4-Cl-IAA- and IAA-treated deseeded pea pericarps over a 24 h experimental period (Johnstone *et al.*, 2005). In this study, we found that 4-Cl-IAA dramatically increased *PsACS1* transcript abundance, with peak transcript levels occurring 2 h after application (1800-fold increase; compare 4-Cl-IAA with SPNS; Fig. 5A; Supplementary Table S3). In contrast, 4-Cl-IAA reduced the transcript abundance of pericarp *PsACS4* (4-fold; Fig. 5C), and minimally affected that of *PsACS2* (Fig. 5B). 4-Cl-IAA’s effect on increasing pericarp *PsACS1* transcript abundance is not likely to be due to 4-Cl-IAA-induced ethylene, as ethephon applied alone or in combination with 4-Cl-IAA had minimal to no effect on *PsACS1* transcript abundance (Fig. 5A; Supplementary Table S3). Additionally, pre-treatment of the pericarp with the ethylene action inhibitor STS did not affect the rise in *PsACS1* transcript abundance 2 h after 4-Cl-IAA treatment; however, this response was attenuated by STS at 8 h and 12 h after hormone treatment, implying that interaction with an activated ethylene signaling pathway prolongs the duration of the 4-Cl-IAA response (Supplementary Fig. S8). A marked increased in ACC (6-fold higher) and ACC-conjugate (2-fold higher) was associated with the elevated *PsACS1* transcript levels in 4-Cl-IAA-treated deseeded pericarps compared with that of the SPNS controls (Figs 2A, B, 6A, B).

IAA increased the *PsACS1* transcript level 2 h after application (~40-fold), but to a much lesser extent than that of 4-Cl-IAA. However, this was only a transitory stimulus as by 8 h after application, the *PsACS1* transcript level in IAA-treated deseeded pericarps was similar to the control (SPNS; Supplementary Table S3). IAA had minor or transitory effects on *PsACS2* and *PsACS4* transcript abundance (Fig. 5B, C). In contrast to 4-Cl-IAA, deseeded pericarps treated with IAA contained 3-fold less ACC and similar ACC conjugate levels compared with SPNS controls (Figs 2A, B, 6A, B).

Subsequent to the stimulation of pericarp *PsACS1* transcript abundance by auxin (2 h after application), both auxins increased pericarp *PsACO1* transcript abundance, 4-Cl-IAA by 11-fold and IAA by ~2-fold (8–12 h after application; Fig. 5D). The increase in *PsACO1* transcript abundance by 4-Cl-IAA was similar in magnitude when 4-Cl-IAA was applied alone or in combination with ethephon or IAA (8 h and 12 h after application; Fig. 5D). However, pre-treatment of the pericarp with the ethylene action inhibitor STS reduced by half the magnitude of the 4-Cl-IAA-induced increase in *PsACO1* transcript abundance (8 h and 12 h after application; Supplementary Fig. S8D), while the IAA-induced increase was slightly reduced by STS pre-treatment 12 h after application (Fig. 5D). These data suggest that *PsACO1* expression in young pea fruit is influenced by auxin and auxin-induced ethylene. Peck and Kende (1995) found that the ethylene action inhibitor 2,5-norbornadiene inhibited the IAA-induced increase in *PsACO1* transcript abundance (using northern analysis) and ACO activity in pea internode tissue, and they concluded that IAA promoted the accumulation of *PsACO1* transcript and the increase in ACO activity through IAA-induced ethylene in this tissue.

4-Cl-IAA suppressed the increase in *PsACO2* and *PsACO3* transcript abundance in deseeded pericarps (compare 4-Cl-IAA with SPNS; Fig. 5E, F), mimicking the presence of the seeds (compare SP with SPNS). Additionally, ethephon treatment did not increase *PsACO2* and *PsACO3* transcript abundance of 4-Cl-IAA-treated pericarps (Fig. 5E, F). As *PsACO2* and *PsACO3* transcript levels were stimulated in deseeded pericarps by ethylene (8–12 h after ethephon application; Fig. 5E, F; Supplementary Fig. S8E, F), and this increase was inhibited by pre-treatment of the pericarp with STS (Supplementary Fig. S8E, F), these data indicate that 4-Cl-IAA may act to reduce ethylene activation of *PsACO2* and *PsACO3* expression. The 4-Cl-IAA-induced changes in the pericarp *PsACO* transcript abundance profile were associated with no change in pericarp ACO enzyme activity (Figs 2A, B, 6C). It is possible that the 4-Cl-IAA-induced pericarp *PsACO* transcript profile (*PsACO1* increased, and *PsACO2* and *PsACO3* decreased) resulted in a pool of ACO proteins that maintained the ACO enzyme activity at levels similar to pericarps with seeds. Additionally, as the ACO protein has also been proposed to have regulatory roles independent of its ability to convert ACC to ethylene (Dilley *et al.*, 2013), it is possible that the function of at least part of the protein varied from that of ACC oxidation to ethylene. Further research on ACO proteins and their levels would aid in determining if the PsACO1 protein has additional regulatory roles during early pea fruit growth and development. It has also been recently proposed that ACC may function as a signaling molecule, independent from its role as a precursor for ethylene biosynthesis (Yoon and Kieber, 2013), in processes such as root cell expansion (Xu *et al.*, 2008) and root response to impaired cell wall biosynthesis (Tsang *et al.*, 2011). If ACC is functioning as a signaling molecule independent from its role as a precursor for ethylene biosynthesis in pea pericarp, 4-Cl-IAA-induced accumulation of ACC may influence processes such as cell expansion in this tissue.

In contrast to 4-Cl-IAA, IAA had transitory to no effects on *PsACO2* and *PsACO3* (Fig. 5E, F). IAA modulation of the *PsACO* transcript abundance pattern was associated with a slightly higher ACO enzyme activity level (50–70%) compared with that in SPNS and 4-Cl-IAA-treated pericarps (Figs 2A, B, 6C). The lower ACC content observed in IAA-treated pericarps is probably due to higher ACO activity in these pericarps compared with the SPNS control (Fig. 6).

The data in this study demonstrate that 4-Cl-IAA and IAA differentially regulate ethylene biosynthesis gene expression. ACC accumulated in 4-Cl-IAA-treated pericarps, driven by high *PsACS1* expression and changes in *PsACO* expression that did not increase ACO enzyme activity, resulting in ACO enzyme activity limiting ethylene production. ACC levels in the IAA-treated pericarps were depleted (compared with SPNS control), due to lower stimulation of *PsACS* gene expression and increased ACO enzyme activity (Fig. 2). Overall, these auxin-induced changes in pericarp *PsACS* and *PsACO* expression probably led to similar whole-pericarp ethylene evolution levels in 4-Cl-IAA- and IAA-treated deseeded pericarps as noted by Johnstone *et al.* (2005). However, we cannot exclude the possibility that ACS protein turnover mediated by ubiquitination and phosphorylation/dephosphorylation (Xu and Zhang, 2015) may also play a role in controlling ACS activity.

### Ethylene signaling during fruit set and ovary senescence

**Fig. 7.**
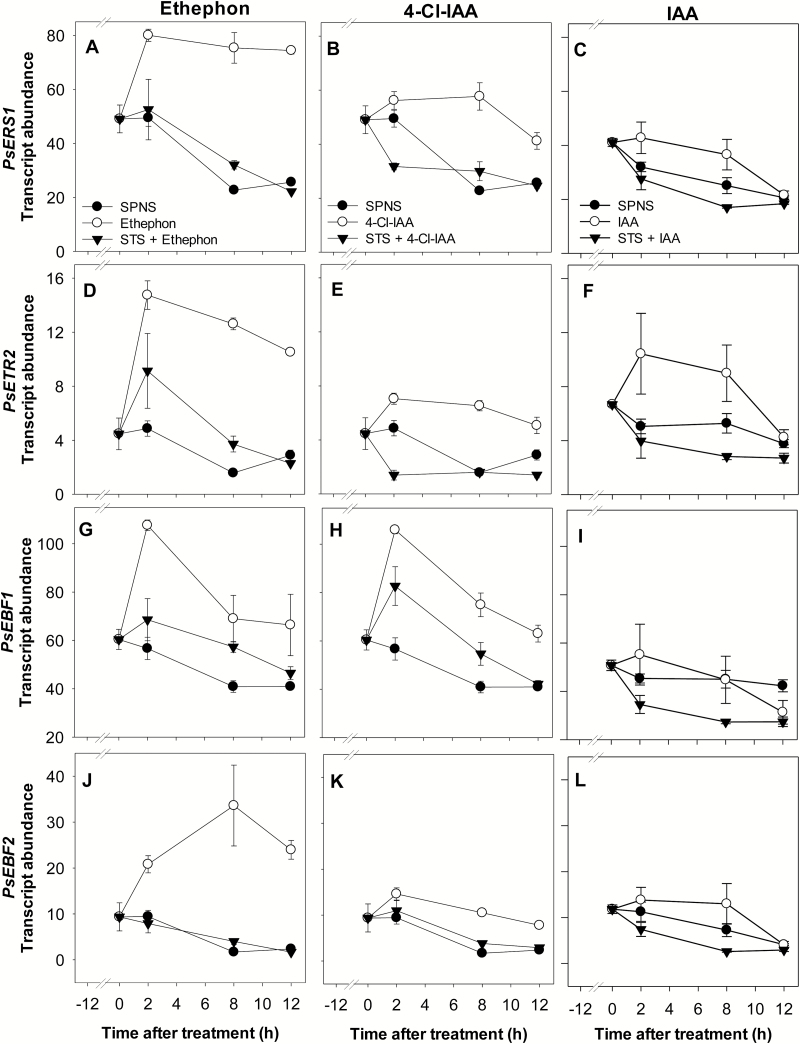
Effect of seed removal and hormone treatment on the relative transcript abundance of the ethylene receptor genes *PsERS1* (A, B, C) and *PsETR2* (D, E, F), and the ethylene signaling-related genes *PsEBF1* (G, H, I) and *PsEBF2* (J, K, L). Two DAA pericarps were split and deseeded (SPNS) and, if STS treated, the deseeded pericarps were immediately treated with STS (1 mM). Twelve hours after splitting and deseeding, pericarps were treated with IAA (50 μM), 4-Cl-IAA (50 μM), or ethephon (1000 mg l^–1^) in 0.1% aqueous Tween-80. SPNS and SP controls were treated with 0.1% aqueous Tween-80. Data are means ±SE, *n*=3–8, with the exception of STS+IAA 2 h treatment, where *n*=2.

In pea pericarps, ethephon application increased the transcript abundance of ethylene receptors *PsERS1* and *PsETR2* (3- to 4-fold after 12 h, Fig. 7A, D), and that of the *PsEBF* genes (the increase in *PsEBF2* transcript levels was most prominent at 18-fold by 8 h; Figs 1C, 7G, J). Pre-treatment of pericarps with STS inhibited the ethephon-induced increase in transcript abundance of the ethylene receptor and signaling-related genes (Fig. 7A, D, G, J) minimally 12 h and 2 h after ethylene treatment, respectively, providing further evidence that these responses were ethylene induced. As ethylene receptors and EBFs act as negative regulators of ethylene signaling (as previously noted in Arabidopsis and tomato; Hua *et al.*, 1998; Kevany *et al.*, 2007; Konishi and Yanagisawa, 2008; Yang *et al.*, 2010), the increased transcript levels of receptor and EBF genes with ethylene treatment in pea fruit indicate that this part of the feedback regulatory mechanisms for damping the ethylene effect probably occurs in pea.

**Fig. 8.**
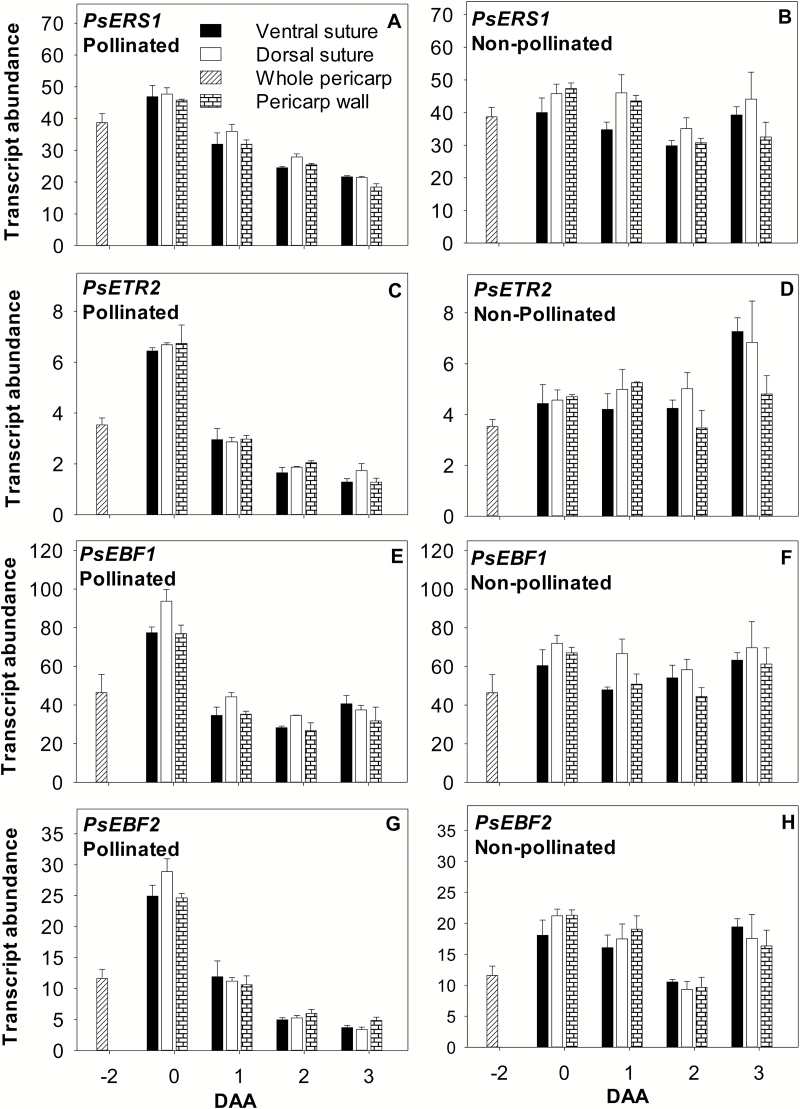
Relative transcript abundance of the ethylene receptor genes *PsERS1* (A and B) and *PsETR2* (C and D), and the signaling-related genes *PsEBF1* (E and F) and *PsEBF2* (G and H) in pollinated and non-pollinated pea fruits. Whole pericarps were assessed at –2 DAA, and pericarp tissues (ventral vascular suture, dorsal vascular suture, and wall) were assessed at 0–3 DAA. For non-pollinated fruits, flowers were emasculated at –2 DAA to prevent pollination. Data are means ±SE; *n*=3, with *n*=2 for a few samples where the tissues were limited due to small tissue size. Each sample is composed of a minimum of four pericarp tissues.

The ethylene receptor (*PsERS1* and *PsETR2*) and signaling-related genes (*PsEBF1* and *PsEBF2*) showed minimal to no spatial variation in their transcript abundance among the pericarp tissues (Fig. 8; Supplementary Table S2). In pollinated pericarps, a gradual reduction in the transcript abundance of *PsERS1*, *PsETR2*, and *PsEBF2* occurred from 0 to 3 DAA, with *PsEBF1* transcript abundance decreasing from 0 to 1 DAA (Fig. 8A, C, E, G), probably reflecting reduced ethylene signaling, as ethylene evolution from the ovaries decreased after pollination/fertilization of the pea ovary (Orzáez *et al.*, 1999). Similarly, transcript levels of the ethylene receptor *NEVER-RIPE* (*NR*; Lashbrook *et al.*, 1998), *SlEBF1*, and *SIEBF2* (Yang *et al.*, 2010) decreased in tomato fruits following pollination. In the absence of pollination, the pericarp transcript abundance of the pea ethylene receptor and EBF genes remained elevated compared with pollinated fruits (Fig. 8), consistent with increased ethylene evolution from non-pollinated fruits (Orzáez *et al.*, 1999) that will undergo senescence.

### Developing seeds and auxin regulation of pericarp ethylene signaling

Compared with intact pericarps, transcript abundance of the ethylene receptor genes, *PsERS1* and *PsETR2*, and the signaling gene *PsEBF2*, was higher in deseeded pericarps 12 h after pericarp splitting and deseeding (0 h after treatment), and their levels declined over the next 12 h to levels nearing that of the intact pericarps (in SP: *PsETR2*, *PsEBF1*, and *PsEBF2* were higher 14 h after pericarp splitting; Supplementary Fig. S9). As the peak of wound ethylene was observed 4 h after pea pericarp splitting (SP) or splitting and seed removal (SPNS), followed by lower ethylene evolution levels in both treatments with time (Johnstone *et al.*, 2005), the general decline in transcript abundance of these ethylene signaling-related genes probably reflects the general decline in ethylene levels (that can up-regulate their expression; Ciardi and Klee, 2001; Konishi and Yanagisawa, 2008; see below) in SP and SPNS over the experimental period.

Application of 4-Cl-IAA to deseeded pericarps increased the transcript abundance of the pericarp ethylene receptors *PsERS1* and *PsETR2* (2.5- and 4-fold increase at 8 h after treatment, respectively; Fig. 7B, E), and the ethylene signaling genes *PsEBF1* and *PsEBF2* (2- and 6-fold increase at 8 h after treatment, respectively; Fig. 7H, K) compared with the SPNS control. IAA treatment had no clear effect on ethylene receptor and *EBF* gene transcript abundance in the pericarp Figs 2C, 7C, F, I, L). Pre-treatment of pericarps with STS inhibited the 4-Cl-IAA-induced increases in transcript abundance of the ethylene receptor and signaling-related genes (Fig. 7B, E, H, K), suggesting that a functional ethylene receptor complex and auxin-induced ethylene are involved in this 4-Cl-IAA response. As 4-Cl-IAA and IAA induced similar ethylene evolution levels over a 24 h period after auxin application to deseeded pea pericarps (Johnstone *et al.*, 2005), the 4-Cl-IAA-specific increase of pericarp ethylene receptor and EBF gene transcript abundance is probably not attributed to higher whole-pericarp ethylene evolution levels in 4-Cl-IAA-treated pericarps. However, we cannot eliminate the possibility that tissue-localized increases in ethylene levels may have contributed to this response.

Turnover and feedback regulation of the ethylene signaling components is complex and includes regulation at the transcriptional, mRNA, and protein turnover levels (Merchante *et al.*, 2013). Our data suggest that increasing pea pericarp ethylene receptor or *EBF* transcript levels may be part of the mechanism that leads to inhibition of ethylene action by 4-Cl-IAA in deseeded pea pericarps, as observed by Johnstone *et al.* (2005).

In summary, previous work demonstrated that the naturally occurring pea auxin 4-Cl-IAA stimulates growth and GA biosynthesis in deseeded pea ovaries, mimicking the presence of seeds; IAA did not (Ozga *et al.*, 2009). Furthermore, although 4-Cl-IAA and IAA stimulate similar pericarp ethylene evolution profiles, 4-Cl-IAA inhibits ethylene response (Johnstone *et al.*, 2005). This study explored the interaction of these auxins with ethylene biosynthesis and signaling pathways to understand how crosstalk between these hormonal pathways can modify ethylene levels and response in the ovary tissue. We found that IAA and 4-Cl-IAA differentially regulated pericarp ethylene biosynthesis gene expression. 4-Cl-IAA stimulated pericarp growth and suppressed the induction of *PsACS4*, *PsACO2*, and *PsACO3* transcript levels in deseeded pericarps (Fig. 2A), mimicking the presence of seeds; IAA did not (Fig. 2A). Auxins, but not ethylene, induced pericarp *PsACS1* transcript levels, with the effect of 4-Cl-IAA being stronger compared with IAA (Fig. 2A), similar to that of the seeds (pericarp *PsACS1* transcript abundance was high in pericarp tissue adjacent to developing seeds, a potential 4-Cl-IAA and IAA source, following pollination; Fig. 1A). 4-Cl-IAA’s marked induction of pericarp *PsACS1* transcript abundance, and its modifications of *PsACO* transcript abundance that were associated with no changes in ACO enzyme activity, led to substantial accumulation of pericarp ACC and ACC conjugate (Fig. 2B). IAA modulated the transcript abundance of the pericarp *PsACS* and *PsACO* genes to a much lesser extent than that of 4-Cl-IAA (Fig. 2A), and the IAA-induced ethylene biosynthesis gene expression profile was associated with a decrease in pericarp ACC content and a modest increase in pericarp ACO enzyme activity (Fig. 2A, B). Overall, these data indicate that ACO activity limited ethylene synthesis in 4-Cl-IAA-treated pericarps, leading to similar ethylene evolution levels in 4-Cl-IAA- and IAA-treated deseeded pericarps observed by Johnstone *et al.* (2005).

With respect to ethylene signaling, 4-Cl-IAA increased the transcript abundance of the pericarp ethylene receptor and signaling-related (*EBF*) genes, whereas IAA did not (Fig. 2C). 4-Cl-IAA-induced increases in pea pericarp ethylene receptor and/or *EBF* transcript abundance (Fig. 2C) may be part of the mechanism involved in the inhibition of ethylene action by 4-Cl-IAA in deseeded pea pericarps (observed by Johnstone *et al.*, 2005).

The interaction of GA with auxin and ethylene has also been explored using the pea split-pericarp system. When applied simultaneously, gibberellin (GA_3_ or GA_1_) and 4-Cl-IAA had an additive effect on pericarp growth (Ozga and Reinecke, 1999); however, simultaneous application of IAA and GA_3_ to deseeded pericarps inhibited GA_3_-stimulated growth (Johnstone *et al.*, 2005). The inhibitory effects of IAA on GA-mediated growth were associated with IAA-induced ethylene and mimicked by ethephon application (Johnstone *et al.*, 2005). GA treatment did not influence the amount of ethylene released from pericarps in the presence or absence of 4-Cl-IAA or IAA (Johnstone *et al.*, 2005). These data suggest that GA stimulates pericarp growth, and ethylene may inhibit GA responses in this tissue. 4-Cl-IAA’s suppression of the growth-inhibiting effects of auxin-induced ethylene on GA-mediated pericarp growth may be partially due to reducing ethylene response, but further experimentation is required to test this hypothesis.

Overall, the data in this study support the working hypothesis that auxin (4-Cl-IAA in pea) produced in the developing seeds stimulates growth and development in the surrounding ovary by regulating a network of hormonal pathways in the ovary including the modulation of ethylene biosynthesis and response.

## Supplementary data

Supplementary data are available at *JXB* online.

Fig. S1. Growth of pollinated and non-pollinated pea fruits (*P. sativum* L. cv. I_3,_ Alaska-type).

Fig. S2. A phylogenetic tree showing the association of pea (*Pisum sativum*) 1-aminocyclopropane-1-carboxylate synthases (PsACSs) within the three ACS protein types of *Arabidopsis thaliana* (At), *Solanum lycopersicum* (Sl), and *Hevea brasiliensis* (Hb) species.

Fig. S3. Alignment of predicted pea ACS sequences with enzymatically active Arabidopsis ACS isozymes.

Fig. S4. A phylogenetic tree showing the association of the predicted pea (*Pisum sativum*) 1-aminocyclopropane-1-carboxylate oxidases (PsACOs) with the ACO proteins of *Arabidopsis thaliana* (At), *Malus domestica* (Md), *Medicago truncatula* (Mt), *Petunia×hybrida* (Ph), *Solanum lycopersicum* (Sl), and *Trifolium repens* (Tr) species.

Fig. S5. Amino acid sequence alignment of the predicted PsACO1, PsACO2, and PsACO3 proteins with those of *Malus domestica* MdACO1, *Petunia hybrida* PhACO, and *Medicago truncatula* Mt5g08533.

Fig. S6. Effect of seed removal and hormone treatment on pea pericarp growth.

Fig. S7. Relative transcript abundance of the ethylene biosynthesis genes *PsACS1* (A), *PsACS2* (B), *PsACS4* (C), *PsACO1* (D), *PsACO2* (E), and *PsACO3* (F) in pericarps of intact, split, or split and deseeded pollinated ovaries.

Fig. S8. Effect of STS pre-treatment on 4-Cl-IAA and ethephon regulation of ethylene biosynthesis gene transcript abundance in deseeded pea pericarps [*PsACS1* (A), *PsACS2* (B), *PsACS4* (C), *PsACO1* (D), *PsACO2* (E), and *PsACO3* (F)].

Fig. S9. Effect of seed removal on the relative transcript abundance of the ethylene receptor genes, *PsERS1* (A) and *PsETR2* (B), and the ethylene signaling-related *EBF* genes, *PsEBF1* (C) and *PsEBF2* (D), in pericarps of pollinated pea ovaries.

Table S1. Primers and probes used for transcript abundance quantitation of pea ethylene biosynthesis, receptor, and signaling-related genes by qPCR, and their PCR efficiencies.

Table S2. Relative transcript abundance of ethylene biosynthesis, receptor, and signaling-related genes in pericarp wall, pericarp dorsal and ventral vascular sutures, and ovules or seeds of pollinated and non-pollinated fruits (1–3 DAA).

Table S3. Effect of seed removal and hormone treatment on *PsACS1* transcript abundance in the pericarps of pollinated pea ovaries.

Table S4. Primers used for the amplification of the full-length CDS of the pea ethylene biosynthesis, receptor, and signaling-related genes.

Supplementary information. Phylogenetic and protein sequence analysis of pea ACSs and ACOs.

Protocol S1. Cloning and sequencing of ethylene biosynthesis and signaling genes.

Protocol S2. qPCR assays.

Protocol S3. Ethylene analysis by gas chromatography.

Protocol S4. Sequence alignment and phylogenetic analysis.

## Author contributions

CPAJ performed the experiments and the data analyses, and provided the initial manuscript draft; KDW provided technical assistance to CPAJ; JAO conceived the project, designed experiments, interpreted the results, and revised the manuscript; DMR conceived the project, contributed to experiments, and revised the manuscript.

## Supplementary Material

Supplementary Figures and Tables
