# Supplementary material for: Regulation of ethylene-related gene expression by indole-3-acetic acid and 4-chloroindole-3-acetic acid in relation to pea fruit and seed development
Source: J Exp Bot. 2017 Jul 11;68(15):4137–51. doi: 10.1093/jxb/erx217 (PMC5853793; doi:10.1093/jxb/erx217)
Supplement: Supplementary Figures and Tables [file erx217_suppl_supplementary_figures_s1_s9_tables_s1_s4_protocols_s1_s4.pdf]

SUPPLEMENTARY DATA

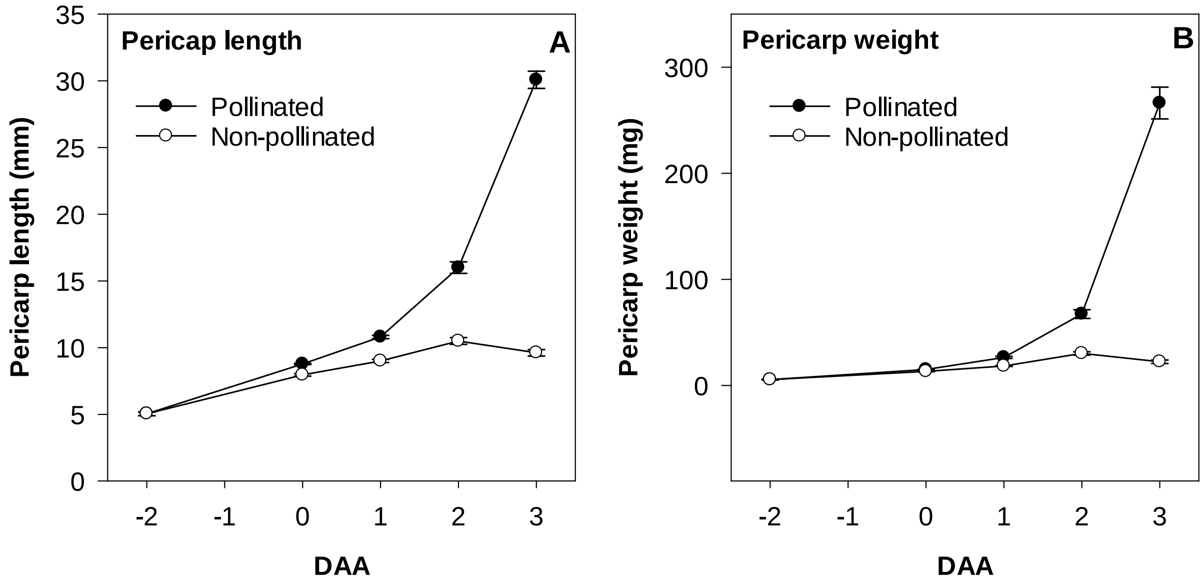

C

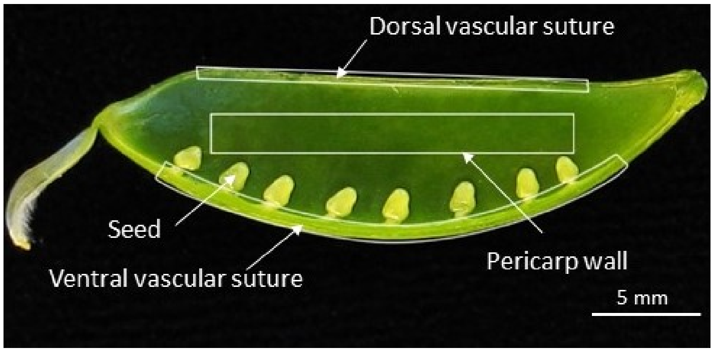

Supplementary Figure S1. Growth of pollinated and non-pollinated pea fruits (*P. sativum L.* cv. I_3, Alaska-type). Pericarp length (**A**) and fresh weight (**B**) of pollinated and non-pollinated pea fruits from -2 to 3 DAA. A representative pollinated pea fruit (ovary; 3 DAA) depicting the pericarp wall, dorsal vascular suture, and ventral vascular suture sections, and seeds, harvested for gene expression analysis (**C**). For the non-pollinated fruits, flowers were emasculated at -2 DAA to prevent pollination. Data are means ± SE; n=12 to 88.

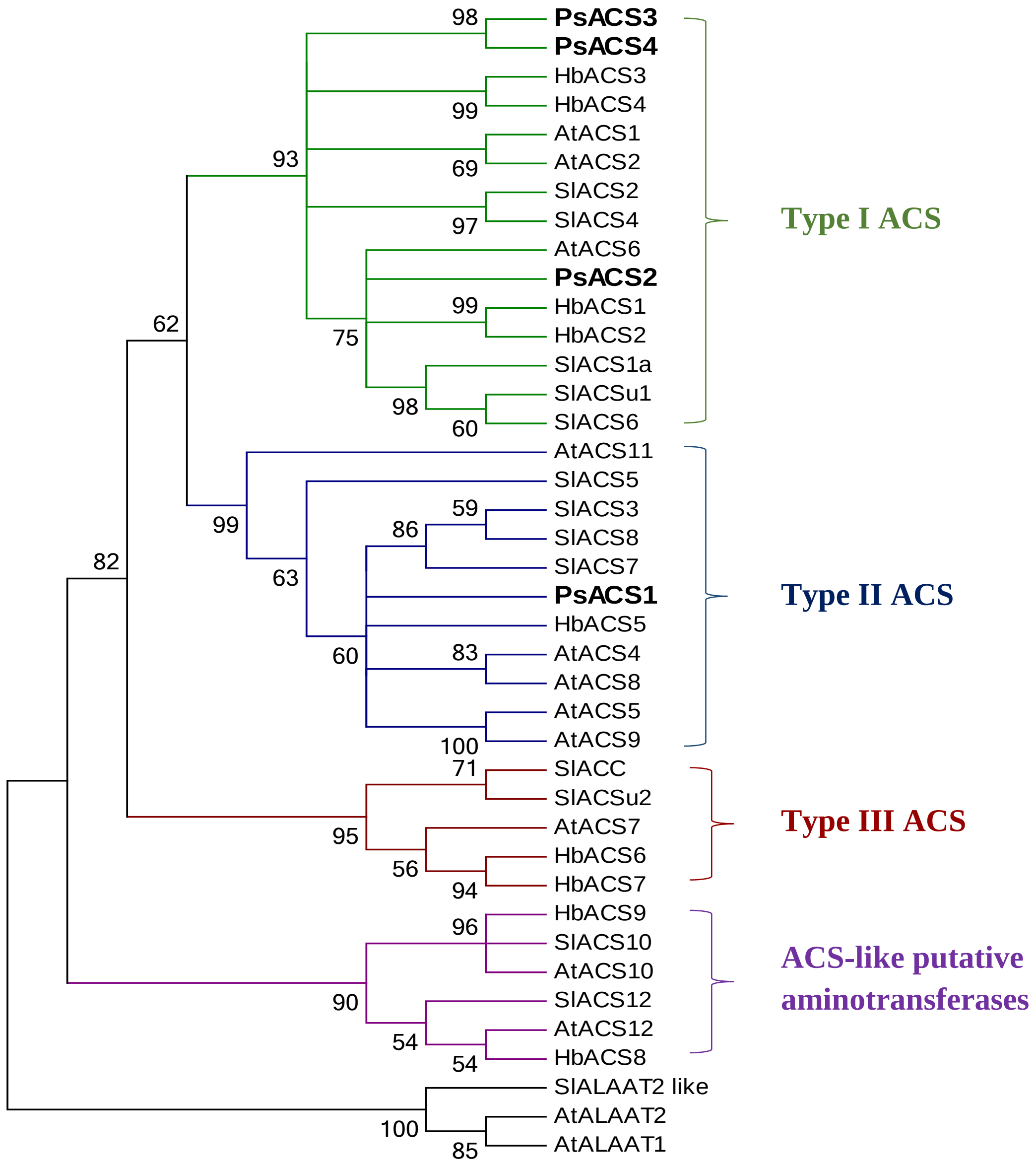

Supplementary Figure S2. A phylogenetic tree showing the association of pea (*Pisum sativum*) 1-aminocyclopropane-1-carboxylate synthases (PsACSs) within the three ACS protein types of *Arabidopsis thaliana* (At), *Solanum lycopersicum* (Sl) and *Hevea brasiliensis* (Hb) species. The amino acid sequence alignment for the phylogenetic tree creation was done using the MUSCLE sequence alignment program under the default settings in MEGA7. A maximum likelihood tree was created with 1000 bootstrap replicates using the LG model with a Gamma distribution (+ G). The tree was rooted with alanine aminotransferase (ALAAT) sequences of Arabidopsis and tomato. Numbers in the branches represent the percentage bootstrap support. Branches with greater than 50% bootstrap support are shown.

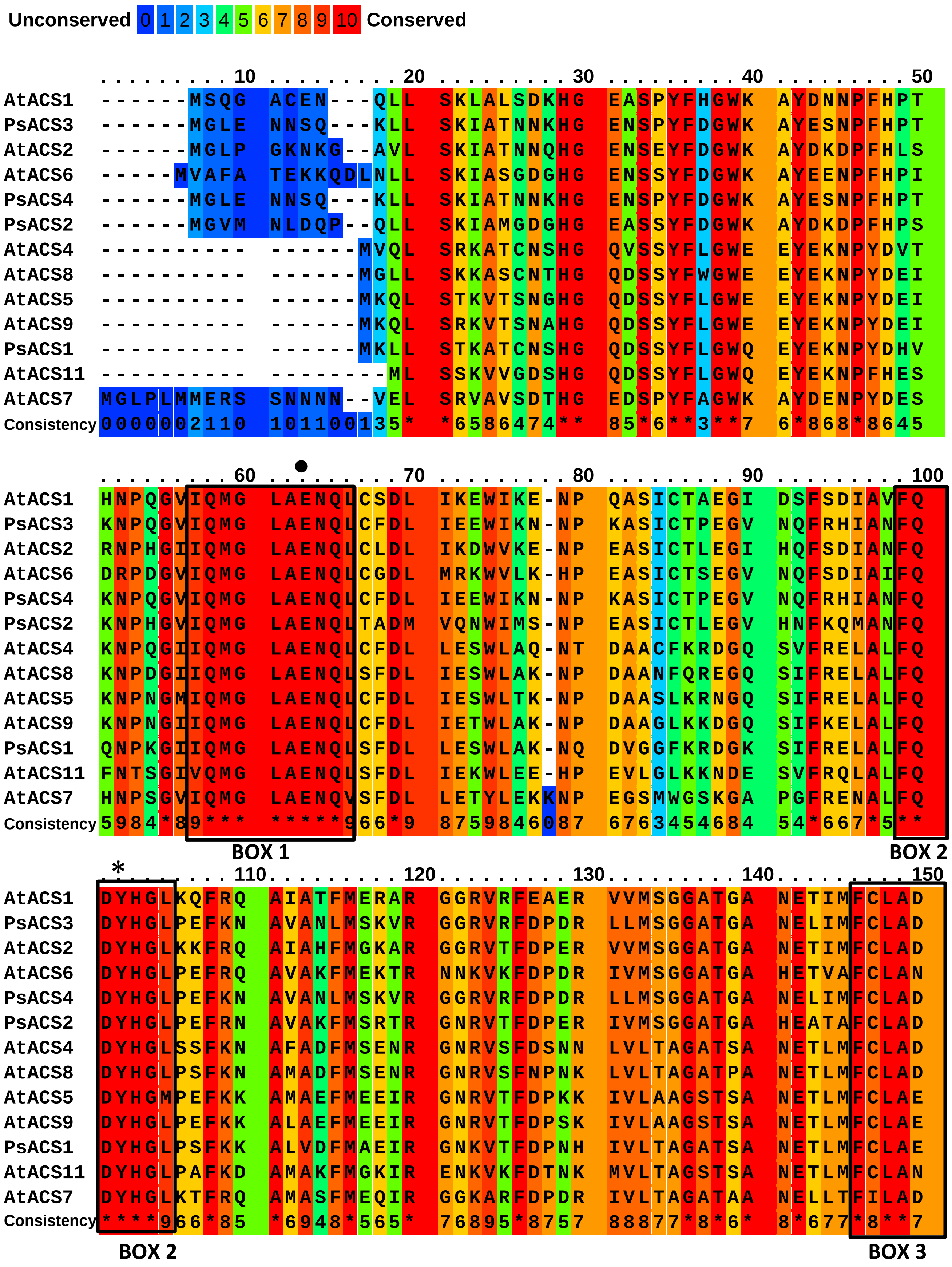
Unconserved 0 1 2 3 4 5 6 7 8 9 10 Conserved
10 20 30 40 50
AtACS1 ------MSQG ACEN---QLL SKLALSDKHG EASPYFHGWK AYDNNPFHPT
PsACS3 ------MGLE NNSQ---KLL SKIATNNKHG ENSPYFDGWK AYESNPFHPT
AtACS2 ------MGLP GKNKG--AVL SKIATNNQHG ENSEYFDGWK AYDKDPFHLS
AtACS6 -----MVAFA TEKKQDLNLL SKIASGDGHG ENSSYFDGWK AYEENPFHPI
PsACS4 ------MGLE NNSQ---KLL SKIATNNKHG ENSPYFDGWK AYESNPFHPT
PsACS2 ------MGVM NLDQP--QLL SKIAMGDGHG EASSYFDGWK AYDKDPFHPS
AtACS4 ---------- ------MVQL SRKATCNSHG QVSSYFLGWE EYEKNPYDVT
AtACS8 ---------- ------MGLL SKKASCNTHG QDSSYFWGWE EYEKNPYDEI
AtACS5 ---------- ------MKQL STKVTSNGHG QDSSYFLGWE EYEKNPYDEI
AtACS9 ---------- ------MKQL SRKVTSNAHG QDSSYFLGWE EYEKNPYDEI
PsACS1 ---------- ------MKLL STKATCNSHG QDSSYFLGWQ EYEKNPYDHV
AtACS11 ---------- --------ML SSKVVGDSHG QDSSYFLGWQ EYEKNPFHES
AtACS7 MGLPLMMERS SNNNN--VEL SRVAVSDTHG EDSPYFAGWK AYDENPYDES
Consistency 0000002110 101100135* *6586474** 85*6**3**7 6*868*8645
60 70 80 90 100
AtACS1 HNPQGVIQMG LAENQLCSDL IKEWIKE-NP QASICTAEGI DSFSDIAVFQ
PsACS3 KNPQGVIQMG LAENQLCFDL IEEWIKN-NP KASICTPEGV NQFRHIANFQ
AtACS2 RNPHGIIQMG LAENQLCLDL IKDWVKE-NP EASICTLEGI HQFSDIANFQ
AtACS6 DRPDGVIQMG LAENQLCGDL MRKWVLK-HP EASICTSEGV NQFSDIAIFQ
PsACS4 KNPQGVIQMG LAENQLCFDL IEEWIKN-NP KASICTPEGV NQFRHIANFQ
PsACS2 KNPHGVIQMG LAENQLTADM VQNWIMS-NP EASICTLEGV HNFKQMANFQ
AtACS4 KNPQGIIQMG LAENQLCFDL LESWLAQ-NT DAACFKRDGQ SVFRELALFQ
AtACS8 KNPDGIIQMG LAENQLSFDL IESWLAK-NP DAANFQREGQ SIFRELALFQ
AtACS5 KNPNGMIQMG LAENQLCFDL IESWLTK-NP DAASLKRNGQ SIFRELALFQ
AtACS9 KNPNGIIQMG LAENQLCFDL IETWLAK-NP DAAGLKKDGQ SIFKELALFQ
PsACS1 QNPKGIIQMG LAENQLSFDL LESWLAK-NQ DVGGFKRDGK SIFRELALFQ
AtACS11 FNTSGIVQMG LAENQLSFDL IEKWLEE-HP EVLGLKKNDE SVFRQLALFQ
AtACS7 HNPSGVIQMG LAENQVSFDL LETYLEKKNP EGSMWGSKGA PGFRENALFQ
Consistency 5984*89*** *****966*9 8759846087 6763454684 54*667*5**
BOX 1
BOX 2
*
110 120 130 140 150
AtACS1 DYHGLKQFRQ AIATFMERAR GGRVRFEAER VVMSGGATGA NETIMFCLAD
PsACS3 DYHGLPEFKN AVANLMSKVR GGRVRFDPDR LLMSGGATGA NELIMFCLAD
AtACS2 DYHGLKKFRQ AIAHFMGKAR GGRVTFDPER VVMSGGATGA NETIMFCLAD
AtACS6 DYHGLPEFRQ AVAKFMEKTR NNKVKFDPDR IVMSGGATGA HETVAFCLAN
PsACS4 DYHGLPEFKN AVANLMSKVR GGRVRFDPDR LLMSGGATGA NELIMFCLAD
PsACS2 DYHGLPEFRN AVAKFMSRTR GNRVTFDPER IVMSGGATGA HEATAFCLAD
AtACS4 DYHGLSSFKN AFADFMSENR GNRVSFDSNN LVLTAGATSA NETLMFCLAD
AtACS8 DYHGLPSFKN AMADFMSENR GNRVSFNPNK LVLTAGATPA NETLMFCLAD
AtACS5 DYHGMPEFKK AMAEFMEEIR GNRVTFDPKK IVLAAGSTSA NETLMFCLAE
AtACS9 DYHGLPEFKK ALAEFMEEIR GNRVTFDPSK IVLAAGSTSA NETLMFCLAE
PsACS1 DYHGLPSFKK ALVDFMAEIR GNKVTFDPNH IVLTAGATSA NETLMFCLAE
AtACS11 DYHGLPAFKD AMAKFMGKIR ENKVKFDTNK MVLTAGSTSA NETLMFCLAN
AtACS7 DYHGLKTFRQ AMASFMEQIR GGKARFDPDR IVLTAGATAA NELLTFILAD
Consistency ****966*85 *6948*565* 76895*8757 88877*8*6* 8*677*8**7
BOX 2
BOX 3

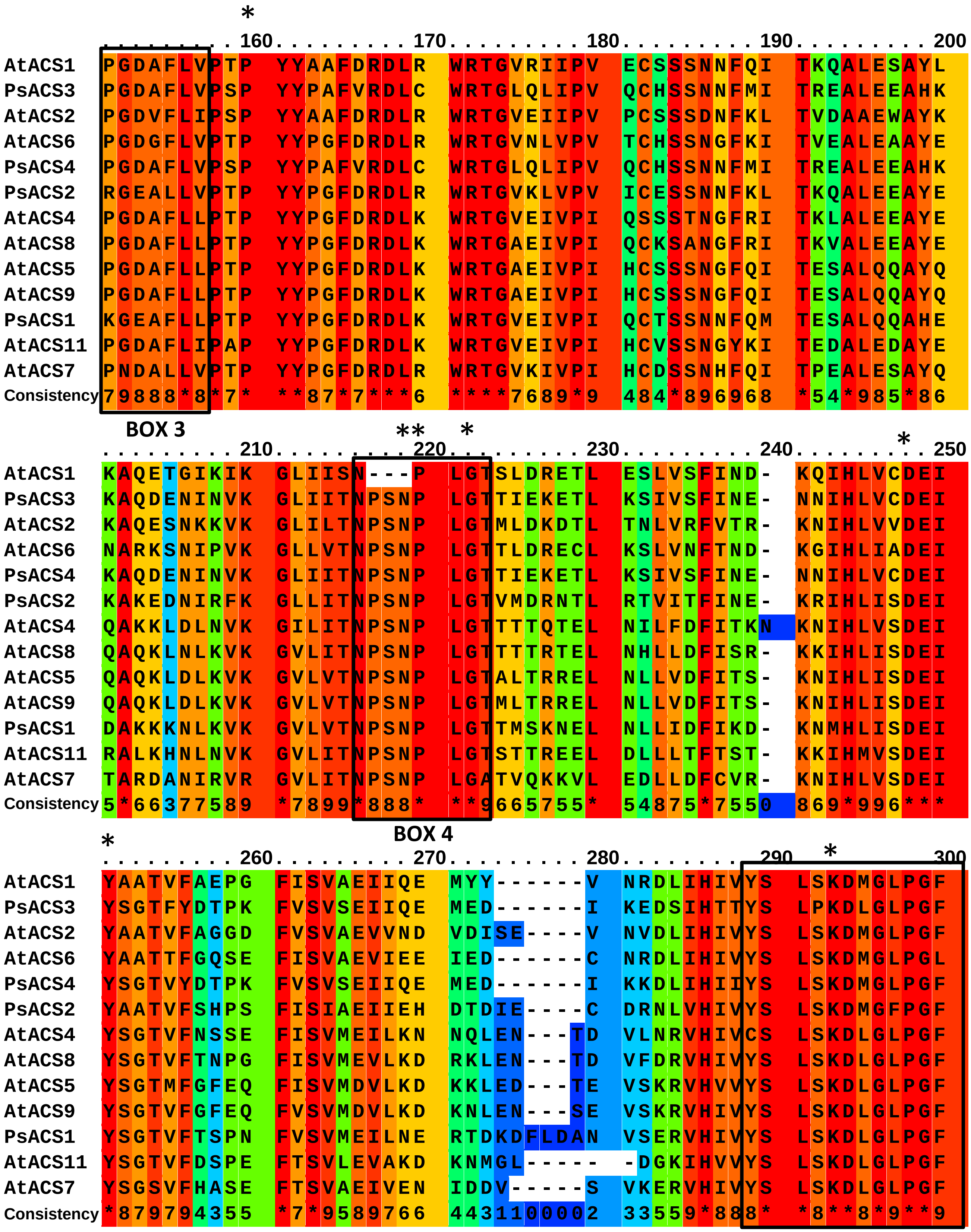
160 170 180 190 200
AtACS1 PGDAFLVPTP YYAAFDRDLR WRTGVRIIPV ECSSSNNFQI TKQALESAYL
PsACS3 PGDAFLVPSP YYPAFVRDLC WRTGLQLIPV QCHSSNNFMI TREALEEAHK
AtACS2 PGDVFLIPSP YYAAFDRDLR WRTGVEIIPV PCSSSDNFKL TVDAAEWAYK
AtACS6 PGDGFLVPTP YYPGFDRDLR WRTGVNLVPV TCHSSNGFKI TVEALEAAYE
PsACS4 PGDAFLVPSP YYPAFVRDLC WRTGLQLIPV QCHSSNNFMI TREALEEAHK
PsACS2 RGEALLVPTP YYPGFDRDLR WRTGVKLVPV ICESSNNFKL TKQALEEAYE
AtACS4 PGDAFLLPTP YYPGFDRDLK WRTGVEIVPI QSSSTNGFRI TKLALEEAYE
AtACS8 PGDAFLLPTP YYPGFDRDLK WRTGAEIVPI QCKSANGFRI TKVALEEAYE
AtACS5 PGDAFLLPTP YYPGFDRDLK WRTGAEIVPI HCSSSNGFQI TESALQQAYQ
AtACS9 PGDAFLLPTP YYPGFDRDLK WRTGAEIVPI HCSSSNGFQI TESALQQAYQ
PsACS1 KGEAFLLPTP YYPGFDRDLK WRTGVEIVPI QCTSSNNFQM TESALQQAHE
AtACS11 PGDAFLIPAP YYPGFDRDLK WRTGVEIVPI HCVSSNGYKI TEDALEDAYE
AtACS7 PNDALLVPTP YYPGFDRDLR WRTGVKIVPI HCDSSNHFQI TPEALESAYQ
Consistency 79888*8*7* **87*7***6 ****7689*9 484*896968 *54*985*86
BOX 3
210 220 230 240 250
AtACS1 KAQETGIKIK GLIISN---P LGTSLDRETL ESLVSFIND- KQIHLVCDEI
PsACS3 KAQDENINVK GLIITNPSNP LGTTIEKETL KSIVSFINE- NNIHLVCDEI
AtACS2 KAQESNKKVK GLILTNPSNP LGTMLDKDTL TNLVRFVTR- KNIHLVVDEI
AtACS6 NARKSNIPVK GLLVTNPSNP LGTTLDRECL KSLVNFTND- KGIHLIADEI
PsACS4 KAQDENINVK GLIITNPSNP LGTTIEKETL KSIVSFINE- NNIHLVCDEI
PsACS2 KAKEDNIRFK GLLITNPSNP LGTVMDRNTL RTVITFINE- KRIHLISDEI
AtACS4 QAKKLDLNVK GILITNPSNP LGTTTTQTEL NILFDFITKN KNIHLVSDEI
AtACS8 QAQKLNLKVK GVLITNPSNP LGTTTTRTEL NHLLDFISR- KKIHLISDEI
AtACS5 QAQKLDLKVK GVLVTNPSNP LGTALTRREL NLLVDFITS- KNIHLISDEI
AtACS9 QAQKLDLKVK GVLVTNPSNP LGTMLTRREL NLLVDFITS- KNIHLISDEI
PsACS1 DAKKKNLKVK GVLVTNPSNP LGTTMSKNEL NLLIDFIKD- KNMHLISDEI
AtACS11 RALKHNLNVK GVLITNPSNP LGTSTTREEL DLLLTFTST- KKIHMVSDEI
AtACS7 TARDANIRVR GVLITNPSNP LGATVQKKVL EDLLDFCVR- KNIHLVSDEI
Consistency 5*66377589 *7899*888* **9665755* 54875*7550 869*996***
BOX 4
260 270 280 290 300
AtACS1 YAATVFAEPG FISVAEIIQE MYY------V NRDLIHIVYS LSKDMGLPGF
PsACS3 YSGTFYDTPK FVSVSEIIQE MED------I KEDSIHTTYS LPKDLGLPGF
AtACS2 YAATVFAGGD FVSVAEVVND VDISE----V NVDLIHIVYS LSKDMGLPGF
AtACS6 YAATTFGQSE FISVAEVIEE IED------C NRDLIHIVYS LSKDMGLPGL
PsACS4 YSGTVYDTPK FVSVSEIIQE MED------I KKDLIHIIYS LSKDMGLPGF
PsACS2 YAATVFSHPS FISIAEIIEH DTDIE----C DRNLVHIVYS LSKDMGFPGF
AtACS4 YSGTVFNSSE FISVMEILKN NQLEN---TD VLNRVHIVCS LSKDLGLPGF
AtACS8 YSGTVFTNPG FISVMEVLKD RKLEN---TD VFDRVHIVYS LSKDLGLPGF
AtACS5 YSGTMFGFEQ FISVMDVLKD KKLED---TE VSKRVHVVYS LSKDLGLPGF
AtACS9 YSGTVFGFEQ FVSVMDVLKD KNLEN---SE VSKRVHIVYS LSKDLGLPGF
PsACS1 YSGTVFTSPN FVSVMEILNE RTDKDFLDAN VSERVHIVYS LSKDLGLPGF
AtACS11 YSGTVFDSPE FTSVLEVAKD KNMGL----- -DGKIHVVYS LSKDLGLPGF
AtACS7 YSGSVFHASE FTSVAEIVEN IDDV-----S VKERVHIVYS LSKDLGLPGF
Consistency *879794355 *7*9589766 4431100002 33559*888* *8**8*9**9

BOX 5

```
           *_*.......310.........320.........330.........340.........350
AtACS1      RVGVVYSYND VVVSCARRMS SFGLVSSQTQ SFLAAMLSDQ SFVDNFLVEV
PsACS3      RVGFVYSSTD EDVDCRTKNS SFGLLRRETQ IFLLQILYDG KTQERFLRAV
AtACS2      RVGIVYSFND SVVSCARKMS SFGLVSSQTQ LMLASMLSDD QFVDNFLMES
AtACS6      RVGIVYSYND RVVQIARKMS SFGLVSSQTQ HLIAKMLSDE EFVDEFIRES
PsACS4      RVGLVYSYND EVVSCGRKMS SFGLVSSQTQ YFLATMLSDD KFMDKFLAES
PsACS2      RVGIIYSYND TVVDCTRKMS SFGLVSTQTQ YLIAKMLSDD DFVEKFLPES
AtACS4      RVGAIYSNDK DVISAATKMS SFGLVSSQTQ YLLSSLLSDK KFTKNYLREN
AtACS8      RVGVIYSNDD FVVSAATKMS SFGLISSQTQ YLLSALLSDK TFTKNYLEEN
AtACS5      RVGAIYSNDE MIVSAATKMS SFGLVSSQTQ YLLSALLSDK KFTSQYLEEN
AtACS9      RVGAIYSNDE MVVSAATKMS SFGLVSSQTQ YLLSALLSDK KFTSTYLDEN
PsACS1      RVGAIYSDNE TVVAAATKMS SFGLVSSQTQ YLLSAMLGDK KFTRNYLSEN
AtACS11     RVGLIYSNNE KVVSAATKMS SFGLISSQTQ HLLANLLSDE RFTTNYLEEN
AtACS7      RVGTIYSYND NVVRTARRMS SFTLVSSQTQ HMLASMLSDE EFTEKYIRIN
Consistency ***59**477 389657698* **8*9989** 579758*7*5 5865589476
```

BOX 5 BOX 6

```
            .........360.........370.........380.........390.........400
AtACS1      SKRVAKRHHM FTEGLEEMGI SCLRS-NAGL FVLMDLRHML KDQ-TFDSEM
PsACS3      QKRLALRREV FTKGTPKVGI KCSQS-YGGL LVCMHLS--L KEK-TVEGEN
AtACS2      SRRLGIRHKV FTTGIKKADI ACLTS-NAGL FAWMDLRHLL RDRNSFESEI
AtACS6      KLRLAARHAE ITTGLDGLGI GWLKA-KAGL FLWMDLRNLL KTA-TFDSET
PsACS4      SRRLRARREF FTKGLEKVNI TCLPS-NAGL FFWMNLRSLL KEK-TFEGEM
PsACS2      AKRLAQRYRV FTGGLIKVGI KCLQS-NGGL FVWMDLRGLL KNA-TFESEI
AtACS4      QKRLKNRQRK LVLGLEAIGI KCLKS-NAGL FCWVDMRPLL RSK-TFEAEM
AtACS8      QIRLKNRHKK LVSGLEAAGI ECLKS-NAGL FCWVDMRHLL KSN-TFEAEI
AtACS5      QKRLKSRQRR LVSGLESAGI TCLRS-NAGL FCWVDMRHLL DTN-TFEAEL
AtACS9      QKRLKIRQKK LVSGLEAAGI TCLKS-NAGL FCWVDMRHLL DTN-TFEAEL
PsACS1      QKRLKKRQKM LVNGLQKAGI SCLKTNNAGL FCWVDMRNLL TSD-TFEAEM
AtACS11     KKRLRERKDR LVSGLKEAGI SCLKS-NAGL FCWVDLRHLL KSN-TFEAEH
AtACS7      RERLRRRYDT IVEGLKKAGI ECLKG-NAGL FCWMNLGFLL EKK-TKDGEL
Consistency 66*953*554 674*85568* 58868078** 957778738* 65409786*5
```

```
            .........410.........420........*430.........440.........450
AtACS1      ALWRVIINKV KINVSPGSSF HCSEPGWFRV CFANMDEDTL QIALERIKDF
PsACS3      DCRV-IVHEV TIIVLPGVSF HCSEPGWYRV CFANMDEETV EIALMRIRAF
AtACS2      ELWHIIIDRV KLNVSPGSSF RCTEPGWFRI CFANMDDDTL HVALGRIQDF
AtACS6      ELWRVIVHQV KLNVSPGGSF HCHEPGWFRV CFANMDHKTM ETALERIRVF
PsACS4      KLWRLIINEV KLNVSPGSAF ECSEPGWYRV CFANMDEETV EIALMRIRAF
PsACS2      ELWRVIIHEV KINVSPGVSF HCSEPGWFRV CYANMDDRDV QIALQRIRSF
AtACS4      DLWKKIVYEV KLNISPGSSC HCEEPGWFRV CFANMIDETL KLALKRLKML
AtACS8      ELWKKIVYEV KLNISPGSSC HCNEPGWFRV CFANLSEETL KVALDRLKRF
AtACS5      DLWKKIVYNV KLNISPGSSC HCTEPGWFRV CFANMSEDTL DLALKRLKTF
AtACS9      ELWKKIVYDV KLNISPGSSC HCTEPGWFRV CFANMSEDTL DLAMKRLKEY
PsACS1      DLWKKILYEV GLNISPGSSC HCTEPGWFRV CFANMSEDTL NLAMKRLKDF
AtACS11     SLWTKIVCEV GLNISPGSSC HCDEPGWFRV CFANMSDQTM EVAMDRVKGF
AtACS7      QLWDVILKEL NLNISPGSSC HCSEVGWFRV CFANMSENTL EIALKRIHEF
Consistency 68854*8469 69898**795 8*5*8**9*9 *9**967687 57*84*8738
```

BOX 7

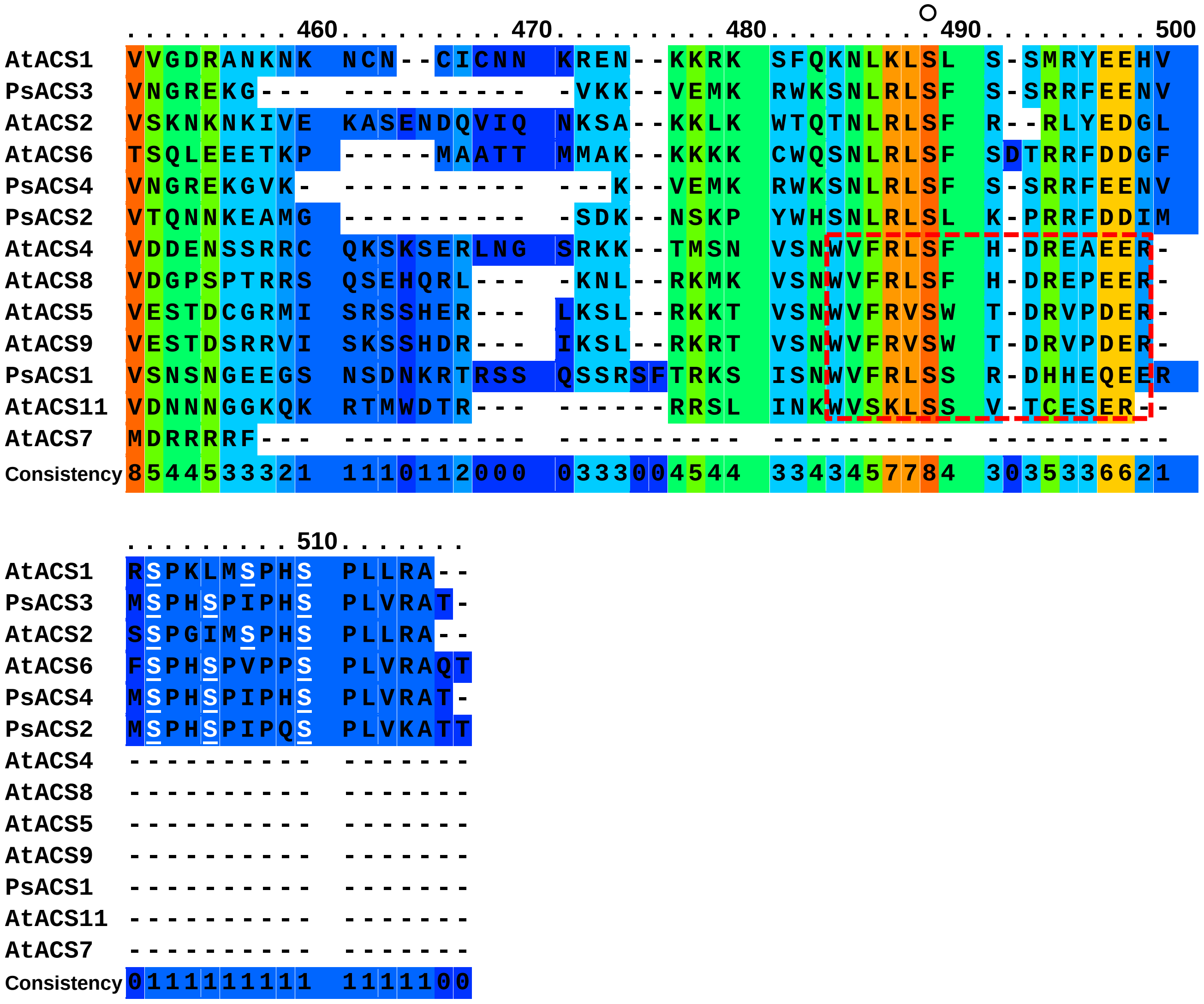

Supplementary Figure S3. Alignment of predicted pea ACS sequences with enzymatically active Arabidopsis ACS isozymes including the AtACS1, which is only active as a heterodimer. Sequences were aligned using the PRALINE multiple sequence alignment tool under default parameters (www.ibi.vu.nl/programs/pralinewww). The seven conserved domains found in the ACS isozymes are marked as boxes 1-7 (Yamagami *et al.*, 2003). The conserved glutamate (E) residue in box 1, which is responsible for substrate specificity, is marked with a solid circle. The amino acids marked with asterisks are conserved among ACS isozymes and also found in various aminotransferases (Yamagami *et al.*, 2003). The CDPK site found only in Type I and Type II ACS is marked with an open circle (Sebastià *et al.*, 2004). The three C-terminal MAPK sites characteristic of type I ACS are marked in white font and underlined (Liu and Zhang, 2004). The C-terminal region with a TOE (Target of ETO1) domain, which is the target site for ETO1 (Ethylene overproducer1) interaction during Type II protein turnover, is marked in a red dashed box (Yoshida *et al.*, 2006). Within the TOE domain, the *PsACS1* sequence contains the conserved WVF sequence and a modified RLSF sequence where F has been substituted with S (AA 484 to 499).

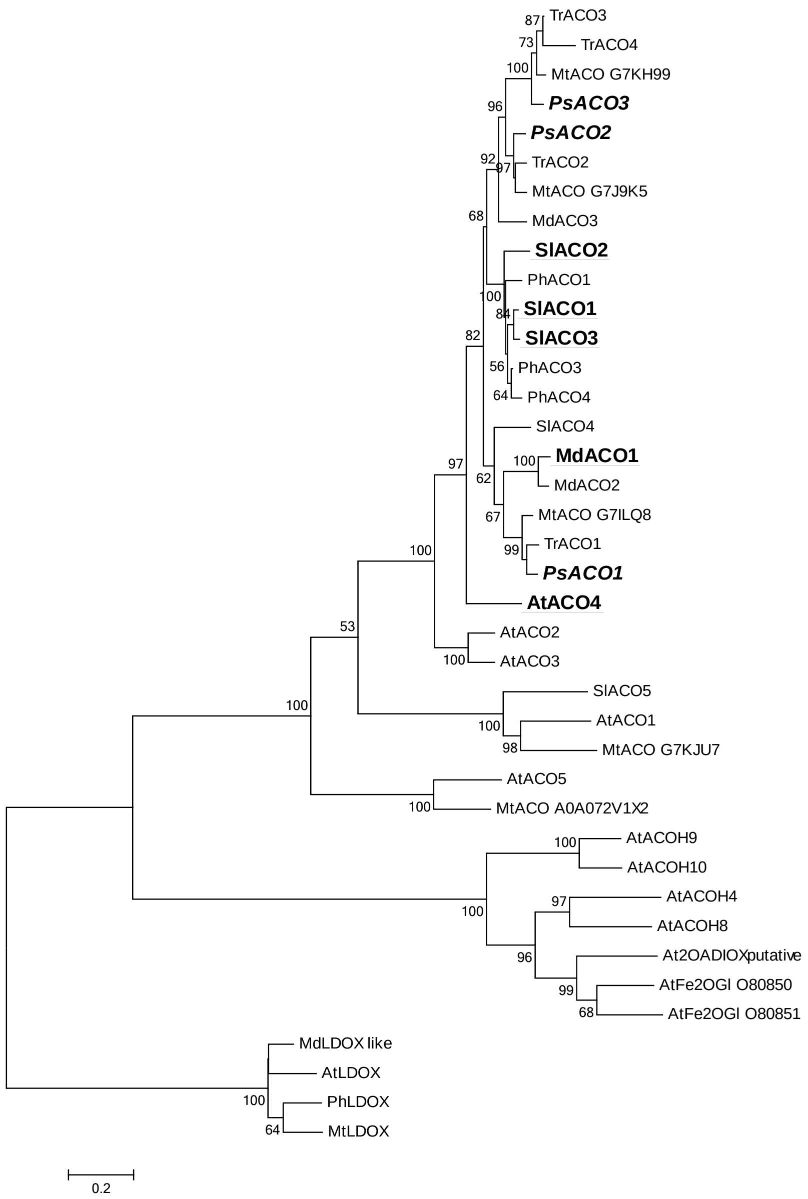

Supplementary Figure S4. A phylogenetic tree showing the association of the predicted pea (*Pisum sativum*) 1-aminocyclopropane-1-carboxylate oxidases (PsACOs) with the ACO proteins of *Arabidopsis thaliana* (At), *Malus domestica* (Md), *Medicago truncatula* (Mt), *Petunia x hybrid* (Ph), *Solanum lycopersicum* (Sl), and *Trifolium repens* (Tr) species. The amino acid sequence alignment for the phylogenetic tree creation was done using the MUSCLE sequence alignment program under the default settings in MEGA7. The maximum likelihood tree was created with 1000 bootstrap replicates using the LG model with a Gamma distribution and invariant sites (G+I); the number of discrete gamma categories was 4. The tree was rooted with leucoanthocyanidin dioxygenase (LDOX) of selected plant species. Bootstrap values with greater than 50% branch support are shown. Pea ACO sequences are in bold and italicized. The ACO proteins of which activity has been verified in vitro (Booker and DeLong, 2015) are in bold and underlined.

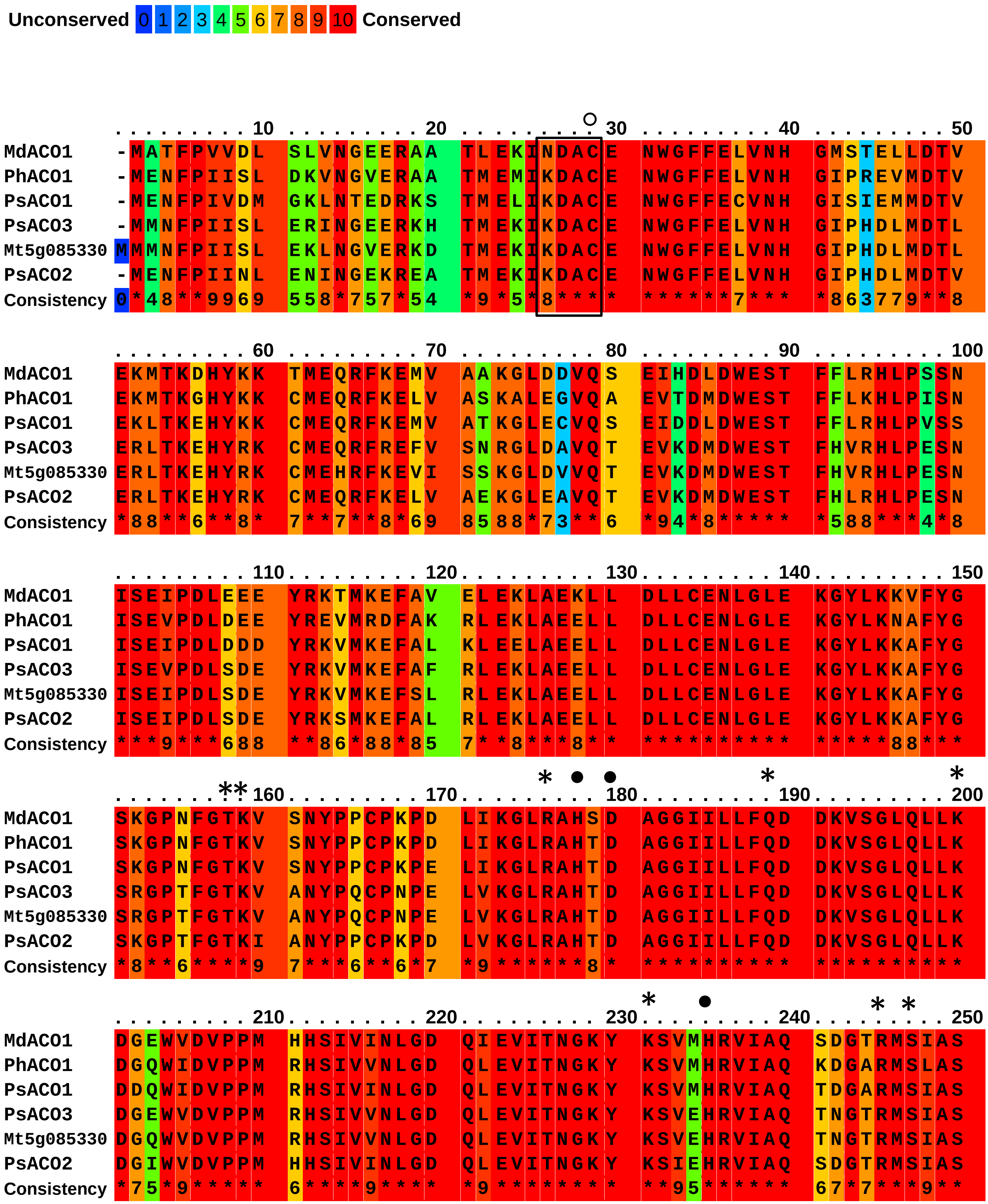
Unconserved 0 1 2 3 4 5 6 7 8 9 10 Conserved
10 20 30 40 50
MdACO1 -MATFPVVDL SLVNGEERAA TLEKINDACE NWGFFELVNH GMSTELLDTV
PhACO1 -MENFPIISL DKVNGVERAA TMEMIKDACE NWGFFELVNH GIPREVMDTV
PsACO1 -MENFPIVDM GKLNTEDRKS TMELIKDACE NWGFFECVNH GISIEMMDTV
PsACO3 -MMNFPIISL ERINGEERKH TMEKIKDACE NWGFFELVNH GIPHDLMDTL
Mt5g085330 MMMNFPIISL EKLNGVERKD TMEKIKDACE NWGFFELVNH GIPHDLMDTL
PsACO2 -MENFPIINL ENINGEKREA TMEKIKDACE NWGFFELVNH GIPHDLMDTV
Consistency 0*48**9969 558*757*54 *9*5*8**** ******7*** *863779**8
60 70 80 90 100
MdACO1 EKMTKDHYKK TMEQRFKEMV AAKGLDDVQS EIHDLDWEST FFLRHLPSSN
PhACO1 EKMTKGHYKK CMEQRFKELV ASKALEGVQA EVTDMDWEST FFLKHLPISN
PsACO1 EKLTKEHYKK CMEQRFKEMV ATKGLECVQS EIDDLDWEST FFLRHLPVSS
PsACO3 ERLTKEHYRK CMEQRFREFV SNRGLDAVQT EVKDMDWEST FHVRHLPESN
Mt5g085330 ERLTKEHYRK CMEHRFKEVI SSKGLDVVQT EVKDMDWEST FHVRHLPESN
PsACO2 ERLTKEHYRK CMEQRFKELV AEKGLEAVQT EVKDMDWEST FHLRHLPESN
Consistency *88**6**8* 7**7**8*69 8588*73**6 *94*8***** *588***4*8
110 120 130 140 150
MdACO1 ISEIPDLEEE YRKTMKEFAV ELEKLAEKLL DLLCENLGLE KGYLKKVFYG
PhACO1 ISEVPDLDEE YREVMRDFAK RLEKLAEELL DLLCENLGLE KGYLKNAFYG
PsACO1 ISEIPDLDDD YRKVMKEFAL KLEELAEELL DLLCENLGLE KGYLKKAFYG
PsACO3 ISEVPDLSDE YRKVMKEFAF RLEKLAEELL DLLCENLGLE KGYLKKAFYG
Mt5g085330 ISEIPDLSDE YRKVMKEFSL RLEKLAEELL DLLCENLGLE KGYLKKAFYG
PsACO2 ISEIPDLSDE YRKSMKEFAL RLEKLAEELL DLLCENLGLE KGYLKKAFYG
Consistency ***9***688 **86*88*85 7**8***8** ********** *****88***
** * • • * *
160 170 180 190 200
MdACO1 SKGPNFGTKV SNYPPCPKPD LIKGLRAHSD AGGIILLFQD DKVSGLQLLK
PhACO1 SKGPNFGTKV SNYPPCPKPD LIKGLRAHTD AGGIILLFQD DKVSGLQLLK
PsACO1 SKGPNFGTKV SNYPPCPKPE LIKGLRAHTD AGGIILLFQD DKVSGLQLLK
PsACO3 SRGPTFGTKV ANYPQCPNPE LVKGLRAHTD AGGIILLFQD DKVSGLQLLK
Mt5g085330 SRGPTFGTKV ANYPQCPNPE LVKGLRAHTD AGGIILLFQD DKVSGLQLLK
PsACO2 SKGPTFGTKI ANYPPCPKPD LVKGLRAHTD AGGIILLFQD DKVSGLQLLK
Consistency *8**6****9 7***6**6*7 *9******8* ********** **********
* • * *
210 220 230 240 250
MdACO1 DGEWVDVPPM HHSIVINLGD QIEVITNGKY KSVMHRVIAQ SDGTRMSIAS
PhACO1 DGQWIDVPPM RHSIVVNLGD QLEVITNGKY KSVMHRVIAQ KDGARMSLAS
PsACO1 DDQWIDVPPM RHSIVINLGD QLEVITNGKY KSVMHRVIAQ TDGARMSIAS
PsACO3 DGEWVDVPPM RHSIVVNLGD QLEVITNGKY KSVEHRVIAQ TNGTRMSIAS
Mt5g085330 DGQWVDVPPM RHSIVVNLGD QLEVITNGKY KSVEHRVIAQ TNGTRMSIAS
PsACO2 DGIWVDVPPM HHSIVINLGD QLEVITNGKY KSIEHRVIAQ SDGTRMSIAS
Consistency *75*9***** 6****9**** *9******** **95****** 67*7***9**

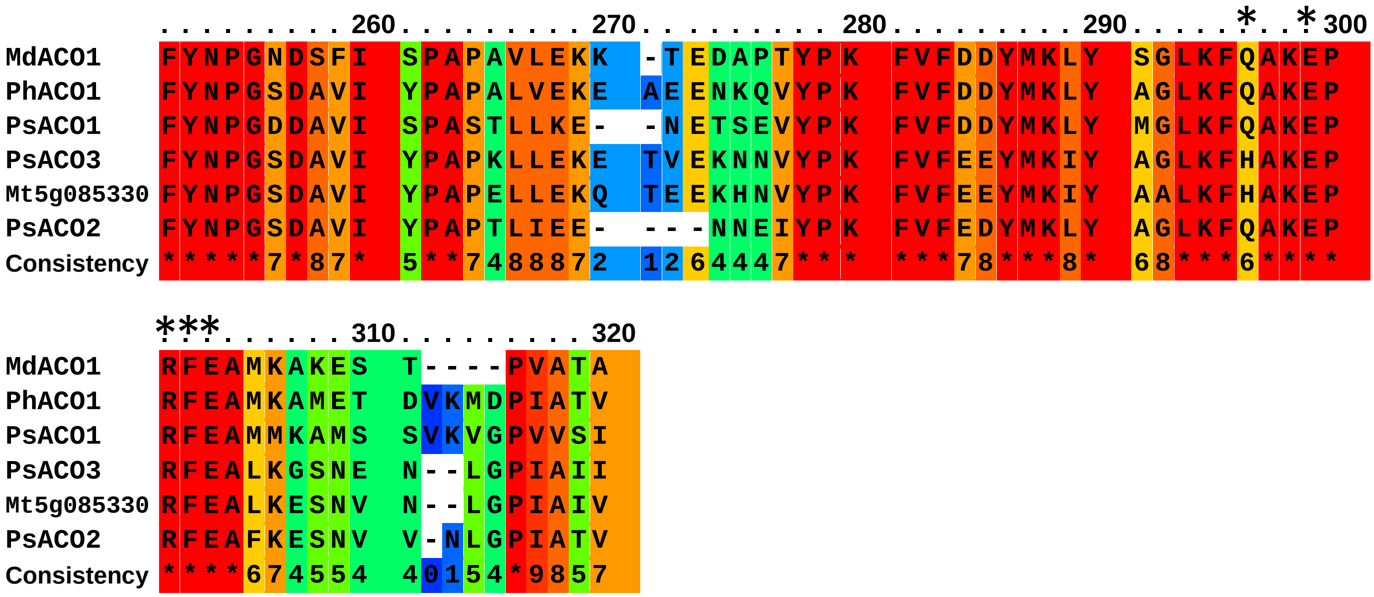

Supplementary Figure S5. Amino acid sequence alignment of the predicted PsACO1, PsACO2 and PsACO3 proteins with those of *Malus domestica* MdACO1 (UniProt ID Q00985), *Petunia hybrida* PhACO1 (UniProt ID Q08506) and *Medicago truncatula* Mt5g085330 (UniProt ID G7KH99). Sequences were aligned using the PSI-PRALINE multiple sequence alignment tool under default parameters (www.ibi.vu.nl/programs/pralinewww). Amino acids shown to be important in ACO enzyme activity include residues involved in the binding of ACC, bicarbonate, and ascorbic acid; these residues are marked with asterisks (Dilley *et al.*, 2013). One modification was noted, a histidine (H) occurs at amino acid (AA) position 296 in PsACO3 instead of a glutamine (Q). However, a homolog of ACO from *Medicago truncatula* also contained an H at AA position 296, suggesting that this variation is not exclusive to PsACO3. The solid black circles mark the amino acid residues involved in Fe (II) binding (Shaw *et al.*, 1996; Zhang *et al.*, 2004). The box with the cysteine residue marked with an open circle represents the putative divalent metal binding site required for cysteine protease activity (Dilley *et al.*, 2013).

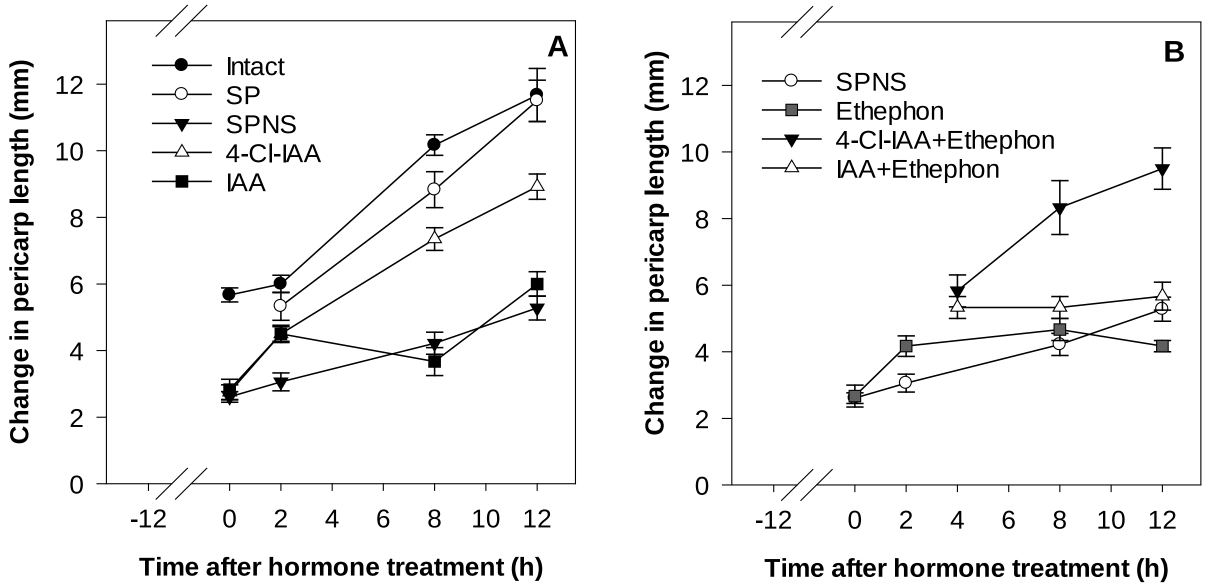

Supplementary Figure S6. Effect of seed removal and hormone treatment on pea pericarp growth (final length minus initial length 12 h after pericarp splitting). Two DAA pericarps were either left intact, split (SP), or split and deseeded (SPNS), and deseeded pericarps were treated with 50 μM IAA or 4-Cl-IAA (**A**), ethephon (1000 mg/L) alone or ethephon in combination with 50 μM IAA or 4-Cl-IAA (**B**). All the hormones were in 0.1% aqueous Tween 80, and treatments were applied 12 h after splitting and deseeding. SPNS and SP controls were treated with 0.1% aqueous Tween 80. When treated with both ethephon and auxin, ethephon was applied 90 min after auxin treatment (with length measurements based on the time of auxin application). Data are means ± SE, n=6 to 18.

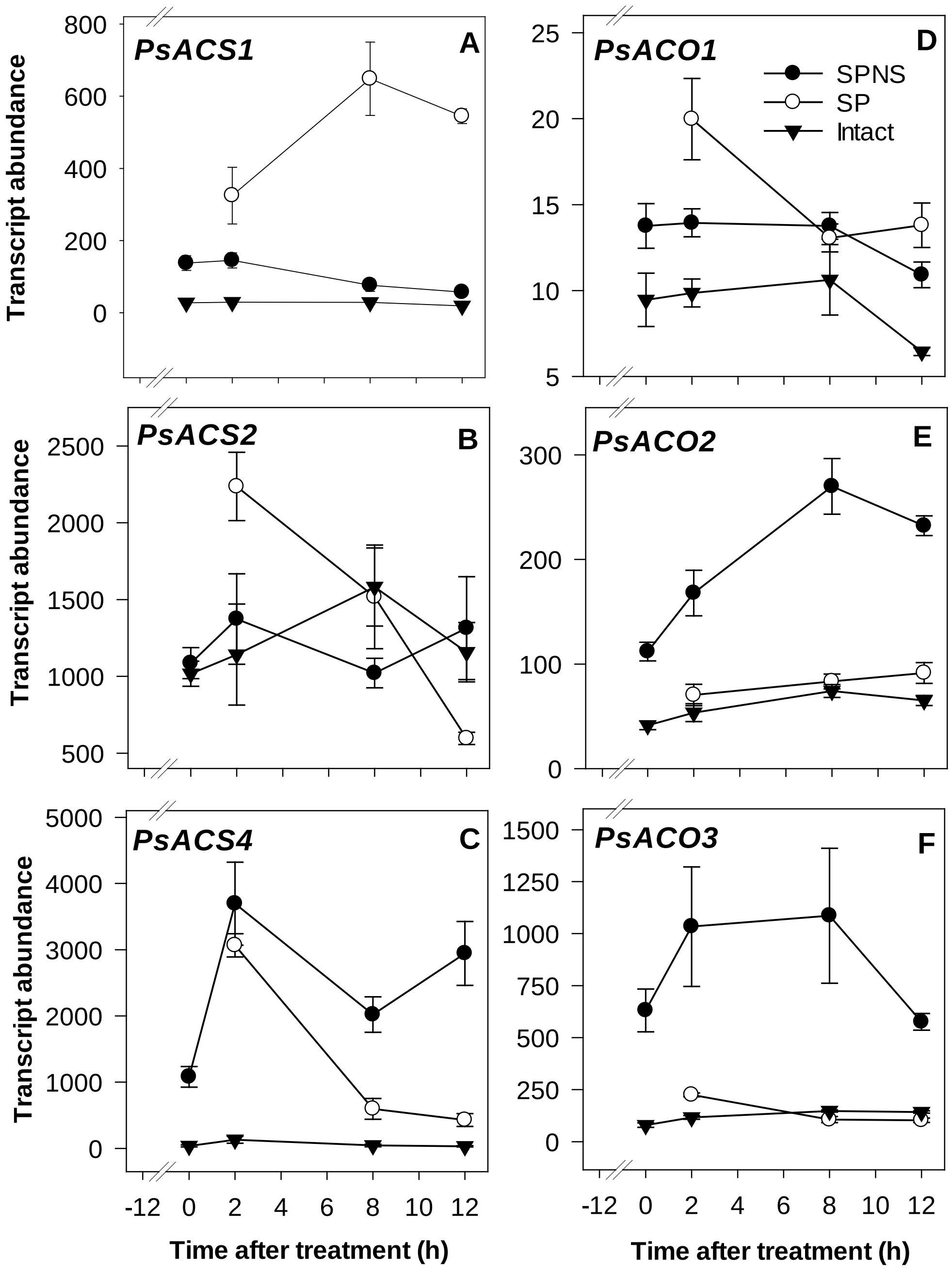

Supplementary Figure S7. Relative transcript abundance of the ethylene biosynthesis genes *PsACS1* (**A**), *PsACS2* (**B**), *PsACS4* (**C**), *PsACO1* (**D**), *PsACO2* (**E**) and *PsACO3* (**F**) in pericarps of intact, split, or split and deseeded pollinated ovaries. Two DAA pericarps were either left intact, split (SP), or split and deseeded (SPNS). An aqueous 0.1% Tween 80 solution was added to the SP or SPNS pericarps 12 h after splitting (at 0 treatment time). Data are means ± SE, n=3 to 8.

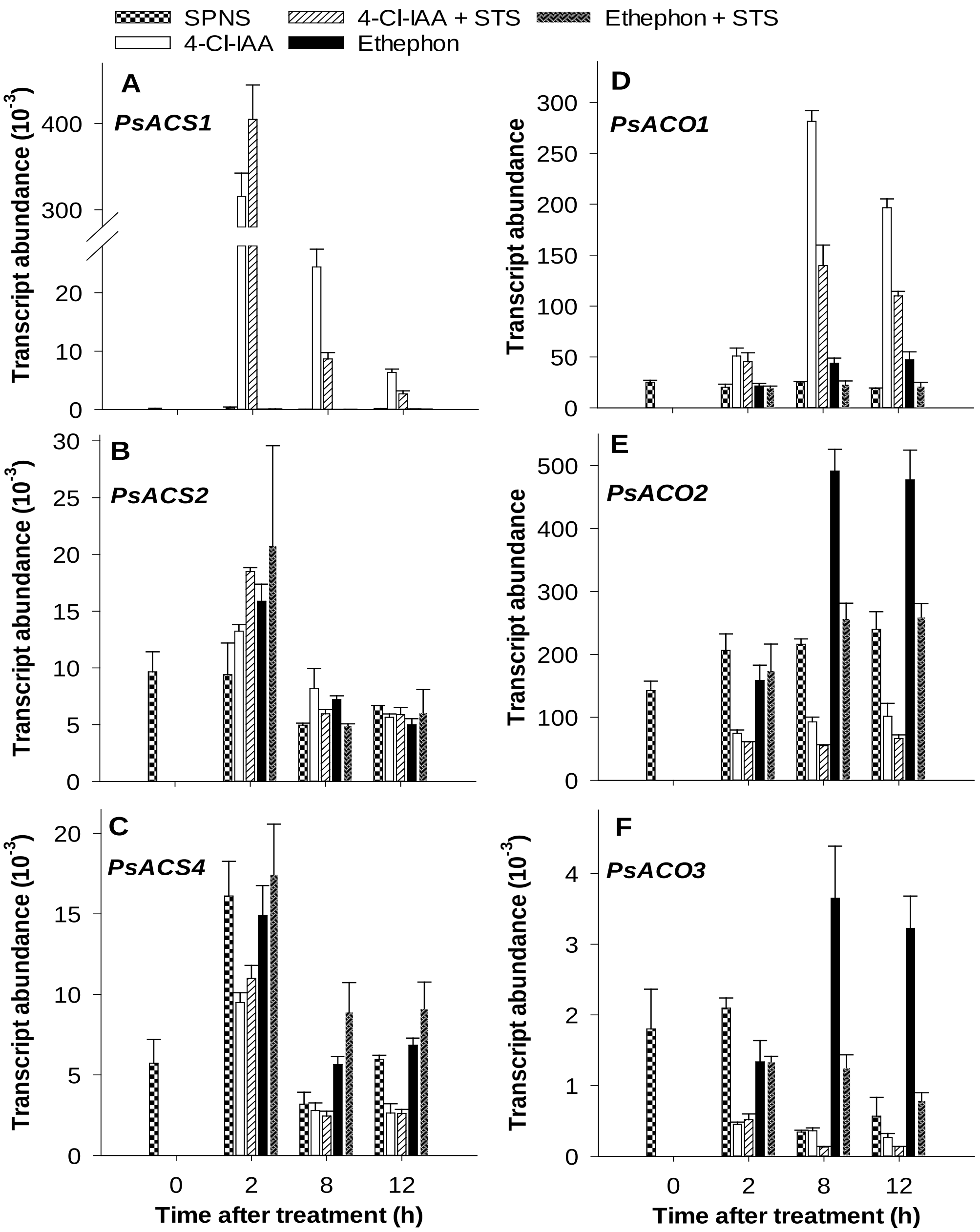

Supplementary Figure S8. Effect of STS pretreatment on 4-Cl-IAA and ethephon regulation of ethylene biosynthesis gene transcript abundance in deseeded pea pericarps [*PsACS1* (**A**), *PsACS2* (**B**), *PsACS4* (**C**), *PsACO1* (**D**), *PsACO2* (**E**) and *PsACO3* (**F**)]. Two DAA pericarps were split and deseeded (SPNS) and immediately treated with STS (1 mM) or they were not treated. Twelve hours after splitting and deseeding, pericarps were treated with 4-Cl-IAA (50 μM) or ethephon (1000 mg L^{-1}) in 0.1% aqueous Tween 80. Data are means ± SE, n=3 (with the exceptions, STS plus ethephon 12 h, SPNS 12 h, ethephon 8 and 12 h, STS plus 4-Cl-IAA 8 h, where n=2).

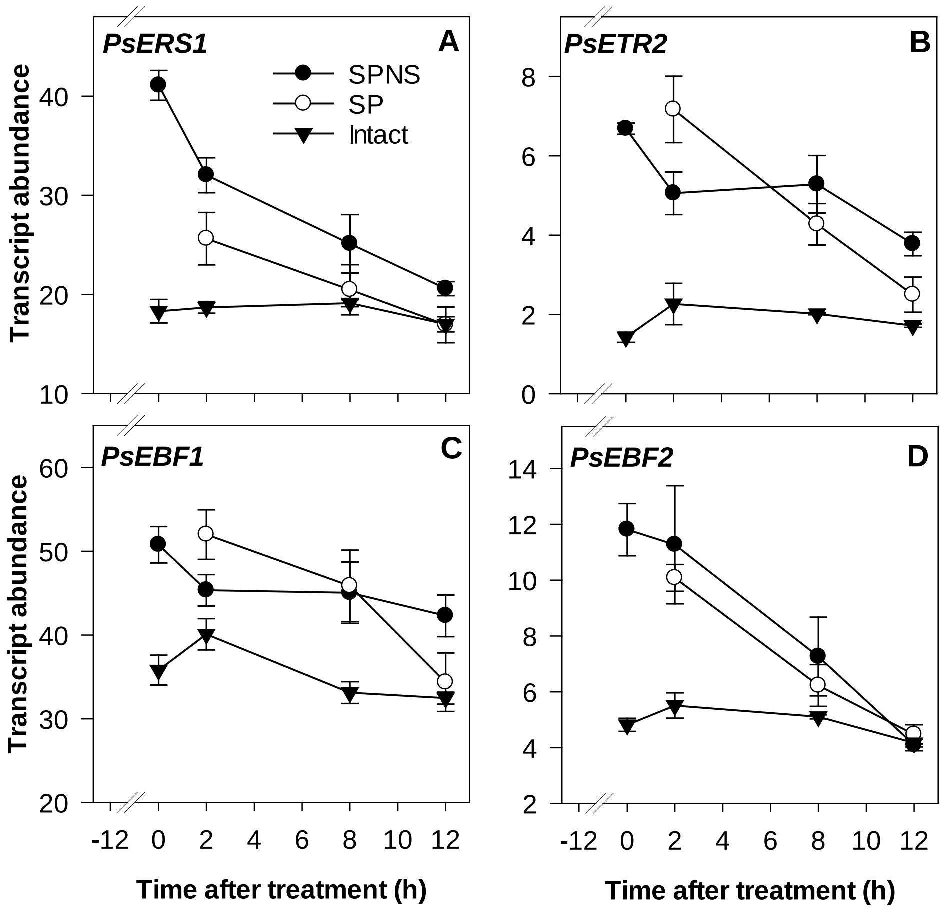

Supplementary Figure S9. Effect of seed removal on the relative transcript abundance of the ethylene receptor genes, *PsERS1* (**A**) and *PsETR2* (**B**), and the ethylene signaling-related *EBF* genes, *PsEBF1* (**C**) and *PsEBF2* (**D**), in pericarps of pollinated pea ovaries. Two DAA pericarps were left intact, split (SP), or split and deseeded (SPNS). An aqueous 0.1% Tween 80 solution was added to the SP or SPNS pericarps 12 h after splitting (at 0 treatment time). Data are means ± SE, n=3 to 8.

Supplementary Table S1. Primers and probes used for transcript abundance quantitation of pea ethylene biosynthesis, receptor, and signaling-related genes by qPCR, and their PCR efficiencies.

Probe-based qPCR assays were used for the quantification of transcript abundance of the pea ethylene biosynthesis (*PsACS1, PsACS2, PsACS4*, *PsACO1*, *PsACO2* and *PsACO3*), ethylene receptor (*PsERS1*and *PsETR2*), and ethylene signaling-related (*PsEBF1* and *PsEBF2*) genes. The probes were labeled with the 6-FAM fluorescent dye at the 5′ end, and double-quenched with Iowa Black FQ (IBFQ) quencher at the 3′ end and the ZEN quencher in the middle (IDT).

Gene	qPCR Efficiency (%)	Primers and Probe	Sequence
PsACS1	93	Forward	CGTTTGAATCCTCCTACATCTTGA
		Reverse	GCAAGAATATGAAAAGAACCCTTATGA
		Probe	TCGGCGAGACCCATCTGAATAATTCCT
PsACS2	94.1	Forward	GCCCGTGAAAACTCTGTACCTT
		Reverse	CGATGATGACTTCGTCGAGAAA
		Probe	CTAACCTCTTCGCACTCTCGGCAAGAAA
PsACS4	100.5	Forward	AGTCTCGTCGCAGACACAATATTT
		Reverse	TGAACTCTCCGCTAAAAACTTATCC
		Probe	TCGCTACAATGCTATCCGATGACAAATTCA
PsACO1	97	Forward	GTGGACTTCAGCTTCTCAAAGATG
		Reverse	CGAGTTGATCACCAAGATTGATG
		Probe	TTGATGTCCCTCCAATGCGTCACTCTATTG
PsACO2	97.2	Forward	CATGTCCAAAACCCGATCTAGTG
		Reverse	TGATGCATGGGTGGAACATCT
		Probe	ACACACCGATGCCGGAGGAATCATC
PsACO3	97	Forward	AATGGAGTGGCGCATTGG
		Reverse	TGGCGGGATCATCCTTCTC
		Probe	AACATCAACCCACTCGCCGTCTTTGA
PsERS1	92.7	Forward	TTGTGGCAATCCAGATTCATCT
		Reverse	GCAAGGCCTCCACTTCCA
		Probe	ATCATCAAGCTACAACCAGAGGCCAAGCA
PsETR2	96.1	Forward	TCTCCAAAGGGAACCTTCCAT
		Reverse	CGAGCAATGTGCTATCAAACTTG
		Probe	TCTTTTGCTGAGCTGTCCATGGCATC
PsEBF1	95.9	Forward	TCAAGGATTGTGCTGGTGTTG
		Reverse	CTGGAGCTTCACCTTTGTCAAA
		Probe	ATCGCTGGCCTCTTTTCTTCAACTTCTTTG
PsEBF2	91.5	Forward	AGACATTGAGGTGGAAGTATCTATGCT
		Reverse	CTTCCGATACCGTGACAGTTTTT
		Probe	TCACCTTGCGAGTCTCTTCGATCTGTAACC

Supplementary Table S2. Relative transcript abundance of ethylene biosynthesis, receptor and signaling-related genes in pericarp wall, pericarp dorsal and ventral vascular sutures, and ovules or seeds of pollinated and non-pollinated fruits (1 to 3 DAA).

Gene	Pollinated				Non-pollinated [a]			
	Seeds	Pericarp wall	Pericarp Dorsal suture	Pericarp Ventral suture	Ovule	Pericarp wall	Pericarp Dorsal suture	Pericarp Ventral suture
PsACS1	696±433 [b]	20.5±7.6	213.3±49.3	417±72.4	134.4± 55.3	6.7±2.5	39.3±13.1	45.3±10.1
PsACS2	725.6±60.3	3152±499	4253±1211	3444±524	1916±537	6351±1035	9639±1480	9794±1595
PsACS4	1080±152	36.4±7.9	76.4±27.5	43.5±10.4	808.2±180.2	461.4±102.2	1221±327	999±354
PsACO1	174.8±22.4	3.6±0.9	38.5±5.5	30.1±7.4	139.5±23.8	7.4±0.9	37±7.0	90±7.1
PsACO2	3.2±1.0	12.6±1.4	50.4±7.0	49.4±4.7	9.8±2.1	44.9±10.0	153.6±47.8	245.3±37.3
PsACO3	45.3±13.2	65.4±14.1	125.9±26.3	93.4±10.7	134±52.9	283.9±85.6	780.3±341.6	741±214.4
PsERS1	50.5±3.4	25.3±2	28.5±2.7	26±1.8	57.1±5.1	35.6±2.5	41.5±3.2	34.6±1.9
PsETR2	7.0±1.2	2.1±0.3	2.2±0.2	2.0±0.3	8.7±1.1	4.5±0.4	5.5±0.5	5.4±0.6
PsEBF1	40.4±1.7	31.2±2.7	38.7±2.0	34.5±2.5	62.7±7.1	52.2±4.0	64.3±4.3	55.9±3.4
PsEBF2	14.7±2.3	7.1±1	6.6±1.5	6.8±1.5	17.6±2.7	15±1.8	14.4±1.9	15.3±1.6

[a] In non-pollinated fruits, flowers were emasculated at -2 DAA to prevent pollination.

[b] Data are means within tissues (harvested 1 to 3 DAA) ± SE, n= 3-4 in ovules/seeds, n= 6-9 in pericarp tissues.

Supplementary Table S3. Effect of seed removal and hormone treatment on *PsACS1* transcript abundance in the pericarps of pollinated pea ovaries.

**Treatment**	**Time after treatment (h)**[a]			
	**0**	**2**	**8**	**12**
**Intact**	27 ± 6[b]	29 ± 6	29 ± 5	19 ± 4
**SPNS**	137 ± 20	145± 21	76 ± 17	57 ± 8
**SP**		324 ± 79	648 ± 102	544 ± 20
**4-Cl-IAA**		269213 ± 19071	11987 ± 2580	3955 ± 1510
**IAA**		5767 ± 1563	93 ± 44	42 ± 8
**Ethephon**		186 ± 41	36 ± 5	102 ± 2
**4-Cl-IAA+Ethephon**			17176 ± 3288	8356 ± 2352
**IAA+Ethephon**			69 ± 21	34 ± 11
**STS+IAA**		5163 ± 3161	25 ± 1	18 ± 3
**STS**		46 ± 10	30 ± 9	20 ± 6
**IAA+4-Cl-IAA**		340795 ± 15682	27275 ± 6891	4912 ± 508

[a] Two DAA pericarps were either left intact, split (SP), or split and deseeded (SPNS) and treated 12 h after deseeding with 4-Cl-IAA (50 μM), IAA (50 μM), or ethephon (1000 mg L^{-1}) in 0.1% aqueous Tween 80 (30 μL) alone or in combination. Auxin or auxin-ethephon combinations were IAA plus 4-Cl-IAA, 4-Cl-IAA plus ethephon, or IAA plus ethephon in 0.1% aqueous Tween 80 (30 μL total). When fruits were treated with both auxin and ethephon, ethephon was applied 90 min after the auxin treatment and samples were collected based on the time after auxin treatment. Because of the delayed ethephon application, the auxin plus ethephon-treated pericarps were not studied at the 2 h time point. Deseeded pericarps were also pretreated with STS (1 mM in 0.1% aqueous Tween 80, 30 μL) at pericarp splitting and deseeding (STS treatment), and IAA (50 μM in 0.1% aqueous Tween 80, 30 μL) was applied to STS pretreated pericarps (IAA plus STS treatment). The SP and SPNS controls were treated with 0.1% aqueous Tween 80 (30 μL). All the samples were collected with respect to the time after hormone treatment.

[b] Data are means ± SE, n=3 to 8, with the exception of STS + IAA 2 h treatment, where n=2.

Supplementary Table S4. Primers used for the amplification of the full-length CDS of the pea ethylene biosynthesis, receptor, and signaling-related genes.

The full-length CDS of the pea ethylene biosynthesis (*PsACS4, PsACO2,* and *PsACO3*), receptor (*PsETR2*), and signaling-related (*PsEBF1* and *PsEBF2*) genes were amplified by PCR using the given primers. The forward primer was designed to bind to the 5′ UTR and the reverse primer to the 3′ UTR of the mRNA, except in the case of *PSACS4*, where the forward primer was designed to bind to a region spanning the 5′ UTR and CDS.

**Gene**	**Primer**	**Sequence**
PsACS3/PsACS4	Forward	CAAGAAATGGGTTTGGAAAA
	Reverse	TGGAAATCCAATTCATCTAGC
PsACO2	Forward	ACCACCTTGTATAGAACTCTAATATGC
	Reverse	AAGGAATAACATCACATCACTCAC
PsACO3	Forward	CAAAAGAGCCAAAAGAAGATAGAA
	Reverse	TGTGTTACCTATTTTCCAAGTACAA
PsETR2	Forward	AGTTGACAGGGCAAGACTGG
	Reverse	TAACAAGAACAAGAATGTGACATGAA
PsEBF1	Forward	CGTCTTCGTTTTCTCTTCGCT
	Reverse	ATTATTGCAGCCATCTAAAAGGC
PsEBF2	Forward	ATGCTCTTCATCCTTTTCTCTCT
	Reverse	ATAAACGATGAACCAAACATGCT

SUPPLEMENTARY INFORMATION

Phylogenetic and protein sequence analysis of pea ACSs and ACOs

The *ACS* genes code for enzymes that catalyze the synthesis of the ethylene precursor ACC. Phylogenetic analysis of the predicted pea ACS proteins clusters them with functional ACS enzymes in *Arabidopsis* (*AtACS2*, *AtACS4*-*AtACS9* and *AtACS11*; Yamagami et al., 2003; Supplementary Fig. S2). Specifically, ACS proteins are divided into three types based on their C-terminal sequence and the presence of putative phosphorylation sites (Xu and Zhang, 2015). Pea ACS proteins PsACS2 and PsACS4 have sequence homology and phosphorylation sites characteristic to Type I ACS (Supplementary Figs. S2 and S3). The Type I ACS genes have a longer C-terminus and contain three mitogen-activated protein kinase (MAPK) phosphorylation sites (Liu and Zhang, 2004) and one calcium-dependent protein kinase (CDPK) site (Sebastià et al., 2004). Phosphorylation of the MAPK and CDPK motifs of type I ACS proteins increases the stability of the protein, which is otherwise rapidly turned over by 26S proteasome-mediated degradation (Booker and DeLong, 2015). Sequence analysis of PsACS1 categorizes it as a Type II ACS. Type II ACS genes have a shorter C-terminus and contain only a putative CDPK site (Supplementary Figs. S2 and S3; Xu and Zhang, 2015). Type II ACS proteins are also degraded through a 26S proteasome-mediated pathway. The E3 ligase component ETHYLENE OVER-PRODUCER 1 (ETO1) or ETO1-like (EOL) proteins interact with the type II ACS at the C-terminal domain known as Target of ETO1 (TOE). The TOE domain consists of the consensus sequence WVF, RLSF and R/D/E rich region which is conserved in numerous species (Yoshida et al., 2006; Booker and DeLong, 2015). The *PsACS1* sequence contains the conserved WVF sequence and a modified RLSF sequence (where F has been substituted with S, see Supplementary Fig. S3, box from AA 484 to 499).

The ACO proteins belong to a large superfamily of ferrous-dependent non-heme oxygenases (Ruduś et al., 2013). Within this family, tomato SlACO1-3, apple (*Malus domestica*) MdACO1 and Arabidopsis AtACO4 have been shown to exhibit ACO activity (Booker and DeLong, 2015). Phylogenetic analysis clusters *PsACO1*, *PsACO2* and *PsACO3* with ACO genes known to code for functional ACC oxidases (Booker and DeLong, 2015; Supplementary Fig. S4). Furthermore, alignment of pea ACO with apple and petunia (*Petunia hybrida*) ACO1 shows that amino acids known to be important in ACO activity are conserved in PsACO1, PsACO2 and

PsACO3 (Supplementary Fig. S5). These results are consistent with the pea ACO genes coding for functional enzymes. One modification was noted, a histidine (H) occurs at amino acid (AA) position 296 in PsACO3 instead of a glutamine (Q). However, a homolog of ACO from *Medicago truncatula* also contained an H at AA position 296, suggesting that this variation is not exclusive to PsACO3 (amino acid positions with respect to the Supplementary Fig. S5). The functions of the conserved AA sequences include binding sites for bicarbonate, ascorbic acid and the Fe (II) cofactor, required for the activation of ACO, and the binding sites for the ACC substrate (Shaw et al., 1996; Zhang et al., 2004; Dilley et al., 2013).

SUPPLEMENTARY PROTOCOLS

Supplementary Protocol S1: Cloning and sequencing of ethylene biosynthesis and signaling genes

Sequences of the putative ethylene biosynthesis and signaling pathway genes *PsACS1*, *PsACS2*, *PsACS3*, *PsACO1* and *PsERS1* were available in the GenBank database (accession numbers AF016458, AF016459, AB049725, M98357 and AF039746.1, respectively). The putative full-length coding sequences of *PsACS4*, *PsETR2*, *PsEBF1* and *PsEBF2* genes were identified using a small scale Next Generation Sequencing database derived from 10 DAA seed coats of *Pisum sativum* L. cvs. I_3 (Alaska-type), Courier, Canstar, Solido and LAN 3017, using a Roche 44 Titanium pyrosequencer at the National Research Council, Saskatoon, Canada. This database consisted of approximately 1.1 million reads with an average length of 336 bp, which was assembled into about 19,000 unigenes (Ferraro, 2014). The putative full-length sequences of *PsACO2* and *PsACO3* were identified using a BLAST search in the NCBI Transcriptome Shotgun Assembly (TSA) database.

For confirmation, full-length coding sequences of *PsACS4*, *PsETR2*, *PsEBF1*, *PsEBF2*, *PsACO2* and *PsACO3* genes were PCR-amplified from pea fruit or seedling tissue cDNA (See Supplementary Table S4 for primers used for PCR). PCR reactions were set up as per the manufacture’s recommendation for PCR amplification. *PsACS4* was PCR amplified using the Qiagen LongRange PCR kit. *PsETR2*, *PsEBF1* and *PsEBF2* were PCR amplified using the Thermo Scientific Phusion Hot Start II DNA Polymerase. *PsACO2* and *PsACO3* were PCR

amplified using the NEB Q5 High-Fidelity DNA Polymerase.

The PCR amplicons obtained were directly used as the template for sequencing of *PsACS4*, *PsEBF1*, *PsACO2* and *PsACO3*. For *PsETR2* and *PsEBF2,* the PCR products were cloned into a pCR8/GW/TOPO vector (Invitrogen) and transformed into chemically competent *E. coli* cells using the pCR8/GW/TOPO TA Cloning Kit (Invitrogen) as per the manufacture's protocol. Plasmids extracted from transformed *E. coli* cultures using GeneJET Plasmid Miniprep Kit (Thermo Scientific) were used for sequencing. All gene sequencing was completed at the Molecular Biology Service Unit, Department of Biological Sciences at the University of Alberta, Edmonton, Alberta, Canada.

The *PsACS1* gene was reported to produce two different mRNAs, *PsACS1a* and *PsACS1b,* due to the presence of an alternate transcription initiation site (Peck and Kende, 1998). As a result, the *PsACS1b* transcript lacks the first 383 nucleotides and encodes a non-functional protein which lacks at least the first 98 amino acids compared to *PsACS1a* (Peck and Kende, 1998). Therefore, to avoid the analysis of non-functional *PsACS1b*, qPCR primers and probes were designed to the 5′ coding region of the mRNA which is unique to *PsACS1a*.

The coding sequence of *PsACS4* (*Pisum sativum* L. cv. I_3 Alaska-type) harbors 98-99% sequence homology to portions of the 5′ and 3′ ends of *PsACS3* (*Pisum sativum* L. var. Saccharatum; GenBank accession number AB049725). However, an approximately 400 bp region (about 800 to 1200 bp from the start codon) was markedly different in *PsACS4* than *PsACS3,* with only 68% similarity at the nucleotide level and 62% similarity at the amino acid level. PCR reactions with cDNA synthesized from total RNA extracted from 6 DAA pericarp wall or 8 DAA seed tissues of *Pisum sativum* cv. I_3 (Alaska-type) produced no target-specific amplicon when using a forward primer that binds to a 5′ region with the same sequence in *PsACS3* and *PsACS4* (5′-CAAGAAATGGGTTTGGAAAA-3′) and a reverse primer specific to the variable region of *PsACS3* (5′-CCACCGTAACTTTGCGAACA-3′). However, the same forward primer produced a target-specific band in PCR reactions when used with a reverse primer that binds to the 3′ region of *PSACS4* that varies from that of *PsACS3* (5′-TGAACTCTCCGCTAAAACTTATCC-3′). Furthermore, primers designed to amplify the full-length sequence of *PsACS3/ PsACS4*, which bind to the identical sequences in the 5′ and 3′ regions of *PsACS3* and *PsACS4* (Supplementary Table S4) amplified only one PCR product from the pea cv. I_3 Alaska-type cDNA. This amplicon had 100% sequence similarity to the

PsACS4 coding sequence. However, as a precaution to ensure that only *PsACS4* transcripts would be targeted, the primers and the probes for *PsACS4* were designed to target the unique region of the *PsACS4* CDS (Supplementary Table S1).

Supplementary Protocol S2: qPCR assays

For all the transcript targets, reverse transcription and quantification were performed as one-step reactions with TaqMan One-Step RT-PCR Master Mix Reagents Kit or TaqMan RNA-to-Ct 1-Step Kit (Applied Biosystems). Each 25 µL reaction contained 200 ng of total RNA (5 µL of 40 ng μL^{-1}), 12.5 µL of 2X master mix, 0.6 µL of 40X MultiScribe/RT Enzyme Mix, 300 nM each of forward and reverse primers (1.5 µL of each 5 µM primer), 100 nM of probe (0.5 µL of 5 µM) and 3.4 µL of nuclease-free water (Ambion). The reactions were performed in MicroAmp Fast Optical 96-well reaction plates covered with MicroAmp Optical Adhesive Film (Applied Biosystems) in a StepOnePlus Real-Time PCR system (Applied Biosystems). The thermal cycler conditions were: 48 ^{o}C for 30 min (reverse transcription), 95 ^{o}C for 10 min (AmpliTaq Gold DNA Polymerase activation), 40 cycles of amplification at 95 ^{o}C for 15 sec (denaturation) and 60 ^{o}C for 1 min (primer annealing and extension). Each sample was run in duplicate and the average of these two technical replicates was used as the sample value. A common pooled total RNA sample was run on each qPCR plate for use as a control to correct for plate to plate variation (Ayele *et al.,* 2006) as follows:

*Normalized Ct value of sample = (Ct value of the common sample in the standard plate / Ct value of the common sample in the sample plate) * Ct value of sample*

The pea 18S small subunit nuclear ribosomal RNA gene was used as a loading control to estimate variation in input total RNA concentration across all samples. For 18S rRNA quantitation, the total RNA samples were diluted to 20 pg μL^{-1} (100 pg total RNA per 25 µL reaction) and assays were performed as described above. The coefficient of variation (CV) of the 18S rRNA Ct among all the samples was 3.0% or lower; therefore, the target amplicon mRNA values were not normalized to the 18S signal (Livak and Schmittgen, 2001; Ozga *et al.,* 2009). The relative transcript abundance was calculated using the $2^{-\Delta Ct}$ method (Livak and Schmittgen, 2001), where ΔCt is the Ct difference of the sample being analyzed and an arbitrary value equal to or greater than the highest assayed Ct value. The arbitrary Ct value was set at 25 for all ethylene signaling genes. For ethylene biosynthesis genes, the arbitrary Ct values were set at 36

for *ACS* genes 28 for *ACO* genes. The efficiency of the qPCR reaction was calculated for each target gene by performing qPCR reactions with a series of total RNA dilutions typically from 150 to 0.015 ng μL^{-1} (or 750 ng to 0.075 ng per reaction) with three technical replicates for each concentration. The Ct values were plotted against the log RNA amount and linear regression (r^2) was calculated. With an acceptable r^2, reaction efficiency (E) was calculated using the formula: $E = (10^{[-1/slope]}-1)*100$ (See Supplementary Table S1 for reaction efficiencies).

Supplementary Protocol S3: Ethylene analysis by gas chromatography

Ethylene from the reaction vial headspace was quantitated using a Varian 3400 Gas chromatograph fitted with a 2.9 m x 6.35 mm HP plot-Q column connected to a flame-ionization detector. The column temperature was isothermal at 35 ^oC, with the injection and detector ports set at 150 ^oC and 250 ^oC, respectively. Helium was used as the carrier gas. Injector head space pressure was set at 20 psi giving a relative retention time for ethylene of 1.2 min. Ethylene standard curves were created by injecting different volumes (generally 10-250 µL) of 10 ppm analytical grade ethylene standard (Praxair, Danbury, USA). The injection volume was 50 µL for free ACC determination, except for 4-Cl-IAA-treated pericarp samples where the injection volume was 20 µL. The injection volumes for total ACC and ACO enzyme activity determination were 100 µL and 200 µL, respectively, for all samples. The number of ethylene moles released was calculated using the ideal gas law considering that the headspace air pressure within the vial remained constant at 101.325 KPa during the reaction.

Supplementary Protocol S4: Sequence alignment and phylogenetic analysis

The amino acid sequence alignments for identification of conserved domains and residues in ACS and ACO were done using the PRALINE multiple sequence alignment application (Simossis and Heringa, 2003; Simossis and Heringa, 2005) under the default settings (gap opening penalty, 12.0; gap extension penalty, 1.0; amino acid substitution matrix, BLOSUM62; alignment strategy, homology-extended alignment). The PRALINE output is presented with color schemes for amino acid conservation as given in the figure legends of Supplementary Figs. S3 and S5.

The amino acid sequence alignment for phylogenetic tree creation for ACS and ACO was done using the MUSCLE sequence alignment program under the default settings in MEGA7. Best model selection and maximum likelihood tree creation with 1000 bootstrap replicates for ACS and ACO were also done in MEGA7. For the ACS tree (Supplementary Fig. S2), the LG model was used with a Gamma distribution (+ G). For the ACO tree (Supplementary Fig. S4), the LG model with a Gamma distribution and invariant sites (G+I) was used (the number of discrete gamma categories was 4). The analysis involved 40 amino acid sequences for the ACS tree and 39 amino acid sequences for the ACO tree. All positions with less than 95% site coverage were eliminated.

The Arabidopsis (*Arabidopsis thaliana*) and tomato (*Solanum lycopersicum*) ACS sequences used in the ACS phylogenetic tree are from Booker and DeLong (2015), and that of *Hevea brasiliensis* from Zhu *et al.* (2015). For the ACO phylogenetic tree, sequences of Arabidopsis, tomato, *Malus domestica,* and *Petunia x hybrida* were from Ruduś *et al.* (2013). The ACO *Medicago* sequences were obtained by a BLAST search of the *Medicago truncatula* Genome Project v4.0 database (http://jcvi.org/medicago/). Uniport accession numbers for the ACO *Medicago* proteins are G7KH99, G7J9K5, G7KJU7, A0A072V1X2, and G7ILQ8. The same Uniport accession numbers have been used for naming the MtACO proteins in the phylogenetic tree.

SUPPLEMENTARY REFERENCES

Ayele BT, Ozga JA, Kurepin L V, Reinecke DM. 2006. Developmental and embryo axis regulation of gibberellin biosynthesis during germination and young seedling growth of pea. Plant Physiology **142**, 1267–1281.

Booker MA, DeLong A. 2015. Producing the ethylene signal: regulation and diversification of ethylene biosynthetic enzymes. Plant Physiology **169**, 42–50.

Dilley DR, Wang Z, Kadirjan-Kalbach DK, Ververidis F, Beaudry R, Padmanabhan K. 2013. 1-Aminocyclopropane-1-carboxylic acid oxidase reaction mechanism and putative post-translational activities of the ACCO protein. AoB Plants. doi: 10.1093/aobpla/plt031

Ferraro KS. 2014. Comparative transcriptomics and proanthocyanidin metabolism in pea

(*Pisum sativum*) seed coat. PhD thesis, University of Calgary.

Liu Y, Zhang S. 2004. Phosphorylation of 1-aminocyclopropane-1-carboxylic acid synthase by MPK6, a stress-responsive mitogen-activated protein kinase, induces ethylene biosynthesis in Arabidopsis. The Plant Cell **16**, 3386–3399.

Livak KJ, Schmittgen TD. 2001. Analysis of relative gene expression data using real-time quantitative PCR and the 2(-Delta Delta C(T)) method. Methods **25**, 402–8.

Ozga JA, Reinecke DM, Ayele BT, Ngo P, Nadeau C, Wickramarathna AD. 2009. Developmental and hormonal regulation of gibberellin biosynthesis and catabolism in pea fruit. Plant Physiology **150**, 448–462.

Peck SC, Kende H. 1998. A gene encoding 1-aminocyclopropane-1-carboxylate (ACC) synthase produces two transcripts: Elucidation of a conserved response. Plant Journal **14**, 573–581.

Ruduś I, Sasiak M, Kępczyński J. 2013. Regulation of ethylene biosynthesis at the level of 1-aminocyclopropane-1-carboxylate oxidase (ACO) gene. Acta Physiologiae Plantarum **35**, 295–307.

Sebastià CH, Hardin SC, Clouse SD, Kieber JJ, Huber SC. 2004. Identification of a new motif for CDPK phosphorylation in vitro that suggests ACC synthase may be a CDPK substrate. Archives of Biochemistry and Biophysics **428**, 81–91.

Shaw J-F, Chou Y-S, Chang R-C, Yang SF. 1996. Characterization of the ferrous ion binding sites of apple 1-aminocyclopropane-1-carboxylate oxidase by site-directed mutagenesis. Biochemical and Biophysical Research Communications **225**, 697–700.

Simossis VA, Heringa J. 2003. The PRALINE online server: optimising progressive multiple alignment on the web. Computational biology and chemistry **27**, 511–519.

Simossis VA, Heringa J. 2005. PRALINE: a multiple sequence alignment toolbox that integrates homology-extended and secondary structure information. Nucleic Acids Research **33** , W289–W294.

Xu J, Zhang S. 2015. Ethylene biosynthesis and regulation in plants. In: Wen C-K, ed. Ethylene in Plants. Springer Netherlands, 1–25.

Yamagami T, Tsuchisaka A, Yamada K, Haddon WF, Harden LA, Theologis A. 2003. Biochemical diversity among the 1-amino-cyclopropane-1-carboxylate synthase isozymes encoded by the Arabidopsis gene family. The Journal of Biological Chemistry **278**, 49102–

49112.

Yoshida H, Wang KL-C, Chang C-M, Mori K, Uchida E, Ecker JR. 2006. The ACC synthase TOE sequence is required for interaction with ETO1 family proteins and destabilization of target proteins. Plant Molecular Biology **62**, 427–437.

Zhang Z, Ren J-S, Clifton IJ, Schofield CJ. 2004. Crystal structure and mechanistic implications of 1-aminocyclopropane-1-carboxylic acid oxidase—The ethylene-forming enzyme. Chemistry and Biology **11**, 1383–1394.

Zhu J-H, Xu J, Chang W-J, Zhang Z-L. 2015. Isolation and molecular characterization of 1-aminocyclopropane-1-carboxylic acid synthase genes in *Hevea brasiliensis*. International Journal of Molecular Sciences **16**, 4136–4149.
